# Supplementary material for: Systematic benchmarking of tools for CpG methylation detection from nanopore sequencing
Source: Nat Commun. 2021 Jun 8;12:3438. doi: 10.1038/s41467-021-23778-6 (PMC8187371; doi:10.1038/s41467-021-23778-6)
Supplement: Supplementary file 1 — Supplementary Information [file 41467_2021_23778_MOESM1_ESM.pdf]

Supplementary Information

Systematic benchmarking of tools for CpG methylation detection from Nanopore sequencing

Zaka Wing-Sze Yuen[1,2], Akanksha Srivastava[1,2], Runa Daniel[3], Dennis McNevin[4], Cameron Jack[2]* & Eduardo Eyras[1,2,5,6]*

[1]EMBL Australia Partner Laboratory Network at the Australian National University, Acton ACT 2601, Canberra, Australia.

[2]The John Curtin School of Medical Research, Australian National University, Acton ACT 2601, Canberra, Australia.

[3]Office of the Chief Forensic Scientist, Victoria Police Forensic Services Department, Macleod 3085, Victoria, Australia.

[4]Centre for Forensic Science, School of Mathematical & Physical Sciences, Faculty of Science, University of Technology Sydney, Australia.

[5]Catalan Institution for Research and Advanced Studies (ICREA), E08010 Barcelona, Spain.

[6]Hospital del Mar Medical Research Institute (IMIM), E08003 Barcelona, Spain.

* co-corresponding authors: cameron.jack@anu.edu.au, eduardo.eyras@anu.edu.au

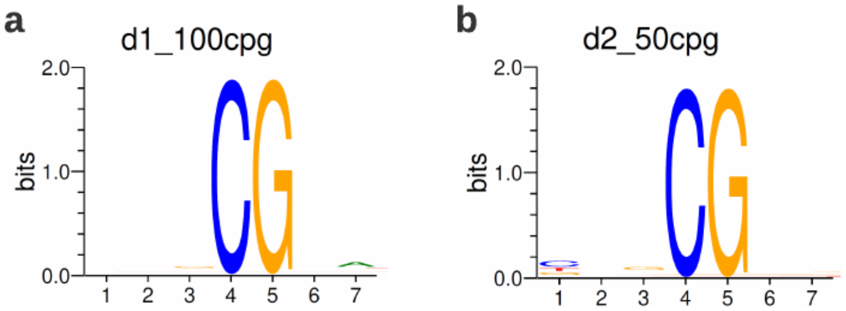

Supplementary Figure 1. Sequence contexts of the CpG sites in datasets 1 and 2. We show the sequence logos with the information content in bits (y axis) for the positions surrounding the CpG sites used in mixture dataset 1 **(a)** and 2 **(b).** The two datasets do not show strong differences in the sequence context around the selected sites.

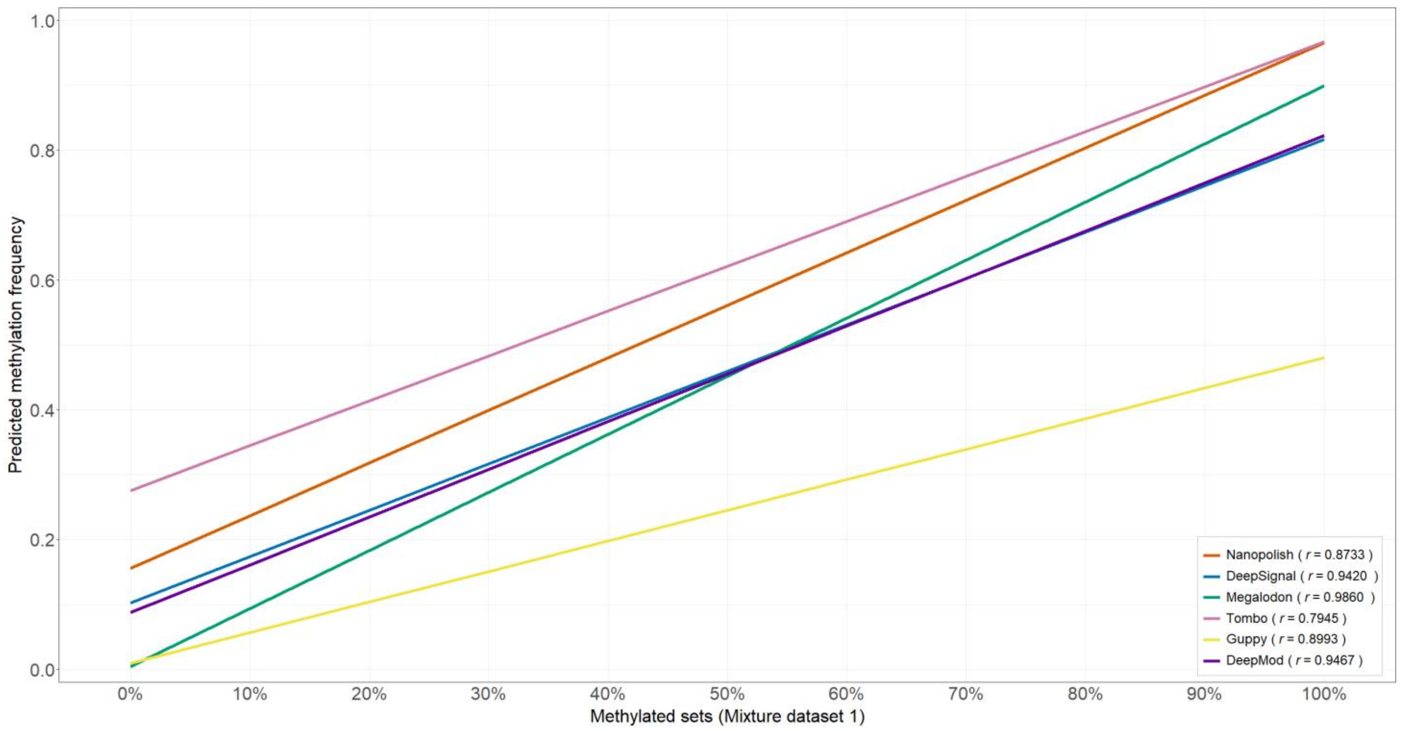

Supplementary Figure 2. Regression lines of the predictions on the mixture dataset 1. This plot uses the same data as Fig. 2a but only showing regression lines and the Pearson's correlation (r) for each tool.

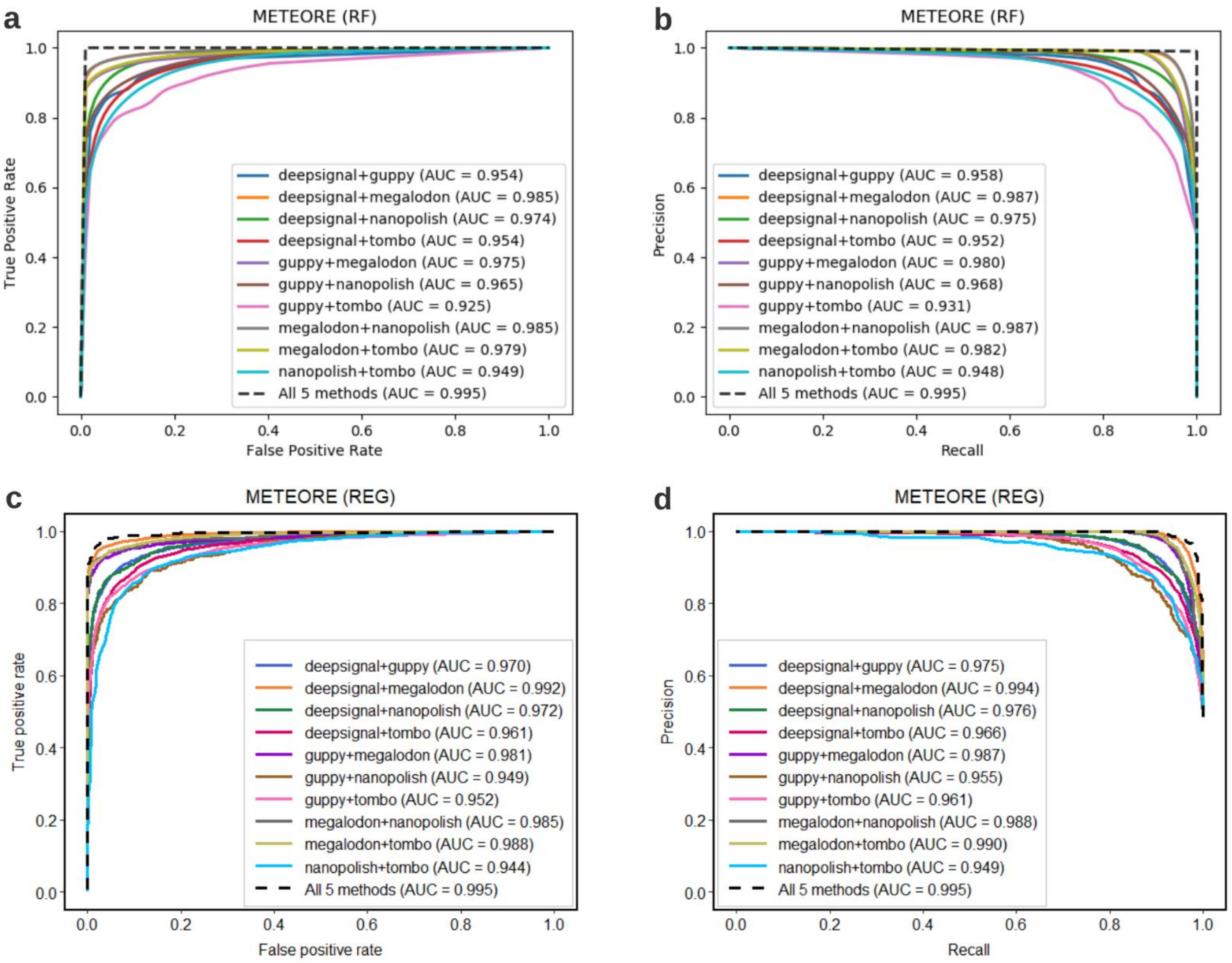

Supplementary Figure 3. Model accuracy for METEORE at the individual read level. (a) Receiver operating characteristic (ROC) curves showing the false positive rate (x axis) and true positive rate (y axis) for the predictions of the METEORE random forest (RF) model combining two methods with default parameters (n_estimator = 100 and max_depth = None) using the sites of the mixture dataset 1 at individual read level. The curves were built from the average of a 10-fold cross validation. **(b)** Precision-recall (PR) curves showing the recall (x axis) and precision (y axis) for the RF model using the sites of the mixture dataset 1 at individual read level, also built from 10-fold cross validation. **(c)** ROC curves for the METEORE regression (REG) model combining two methods at individual read level. Curves were built from a 5-fold cross validation using the sites of mixture dataset 1. **(d)** PR curves for the METEORE REG model using the sites of the mixture dataset 1 at individual read level, built from a 5-fold cross validation.

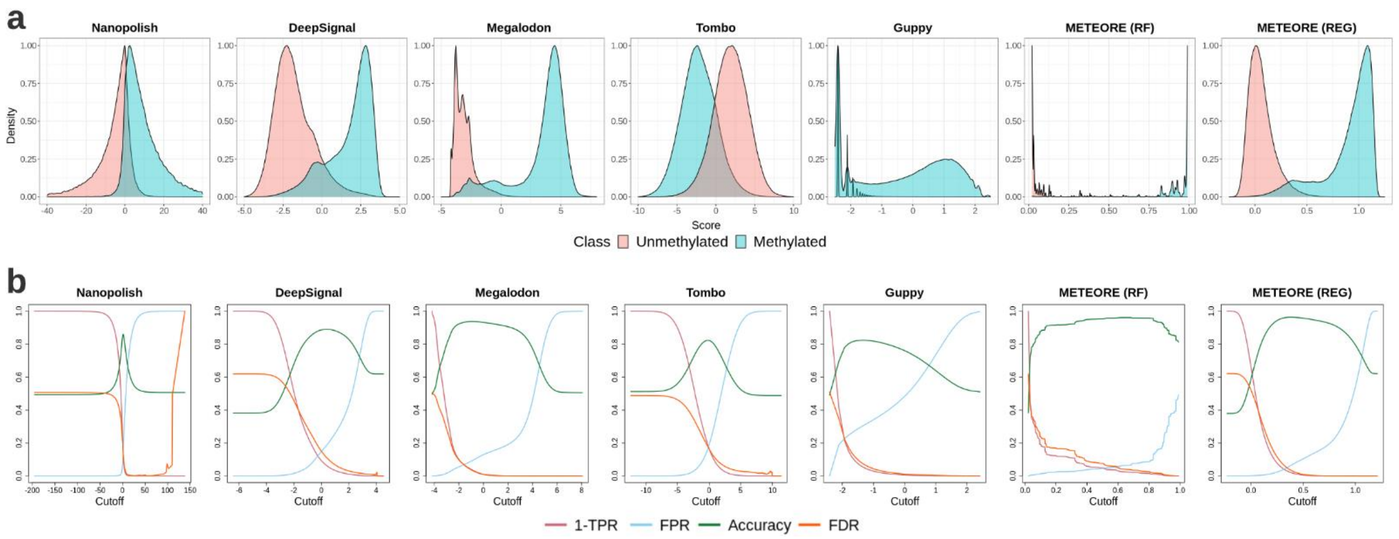

Supplementary Figure 4. Score distributions and accuracy metrics. (a) Score (x axis) distribution for methylated and unmethylated sites in individual reads for each tested tool on the mixture dataset 1, including METEORE. We used METEORE combining the predictions of Megalodon and DeepSignal with a random forest (RF) (parameters: max_dep=3 and n_estimator=10) and with a regression model (REG). **(b)** Distribution of various accuracy metrics (y axis) according to the score (x axis) for each method shown in **(a)**. We show the false positive rate (FPR), false discovery rate (FDR), 1 - true positive rate (TPR), and Accuracy curves as a function of the single score (x axis) cutoff for each tool, where FPR = FP/(FP+TN), TPR = TP/(TP+FN), FDR = FP/(TP+FP), accuracy = (TP + TN)/(TP + TN + FP + FN); and TP = true positives, FP = false positives, TN = true negatives, and FN = false negatives.

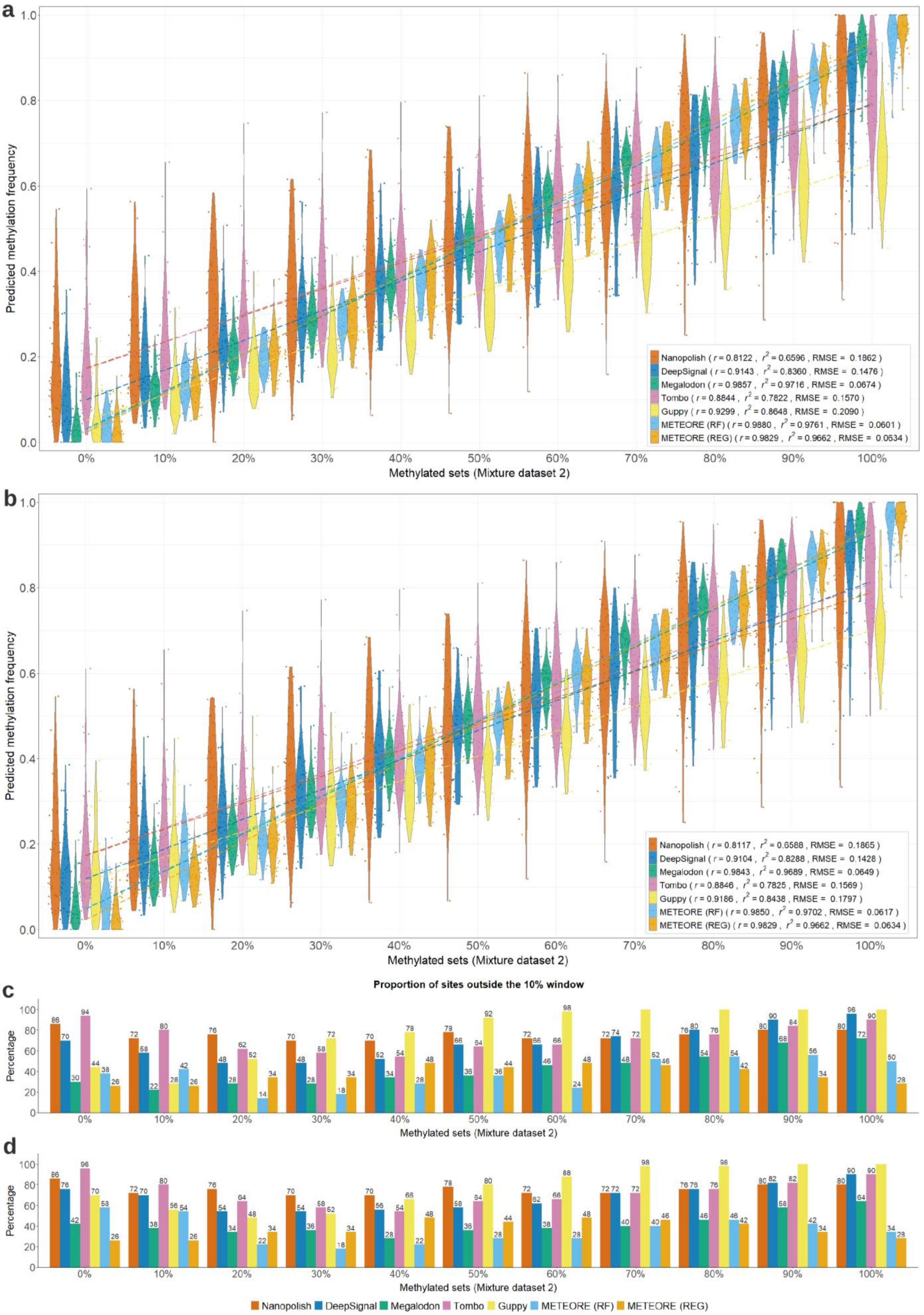

Supplementary Figure 5. Accuracy analysis of five individual tested tools using a single score cutoff. (a-b) Violin plots showing the predicted methylation frequencies (y axis) for each control mixture set with a given proportion of methylated reads (x axis) from the mixture dataset 2 for the five tested tools plus

METEORE combining Megalodon and DeepSignal using random forest (RF) and regression (REG) models with the single threshold obtained by **(a)** the maximum value of (TPR-FPR) or **(b)** the minimum value of $FPR^2 + (1-TPR)^2$. Score thresholds are given in Supplementary Table 3. The Pearson's correlation (r), coefficient of determination (r^2) and the root mean square error (RMSE) are given for each tool. **(c-d)** Barplot showing the proportion of sites predicted outside a 10% window around the expected methylation proportion for each method with the single threshold obtained by **(c)** the maximum value of (TPR-FPR) or **(d)** the minimum value of $FPR^2 + (1-TPR)^2$. Each predicted site in the m% dataset was classified as "outside" if its predicted percentage methylation was outside the interval [(m-5)%, (m+5)%] for intermediate methylation values, or outside the intervals [0,5%] or [95%,100%] for the fully unmethylated or fully methylated sets, respectively. TPR = true positive rate, FPR = false positive rate.

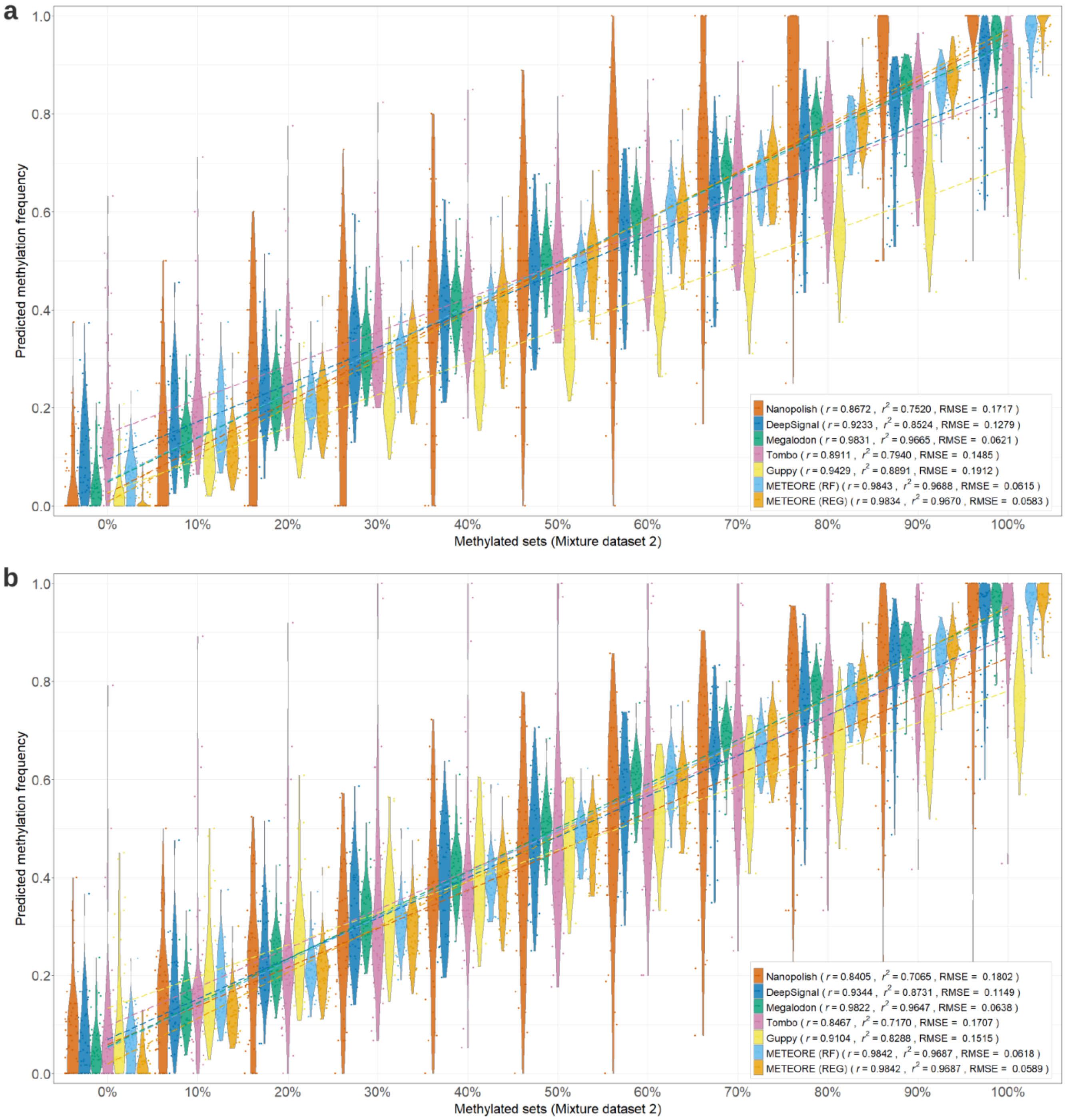

a
Predicted methylation frequency
0.0
0.2
0.4
0.6
0.8
1.0
0%
10%
20%
30%
40%
50%
60%
70%
80%
90%
100%
Methylated sets (Mixture dataset 2)
Nanopolish (r = 0.8672 , r2 = 0.7520 , RMSE = 0.1717)
DeepSignal (r = 0.9233 , r2 = 0.8524 , RMSE = 0.1279)
Megalodon (r = 0.9831 , r2 = 0.9665 , RMSE = 0.0621)
Tombo (r = 0.8911 , r2 = 0.7940 , RMSE = 0.1485)
Guppy (r = 0.9429 , r2 = 0.8891 , RMSE = 0.1912)
METEORE (RF) (r = 0.9843 , r2 = 0.9688 , RMSE = 0.0615)
METEORE (REG) (r = 0.9834 , r2 = 0.9670 , RMSE = 0.0583)
b
Predicted methylation frequency
0.0
0.2
0.4
0.6
0.8
1.0
0%
10%
20%
30%
40%
50%
60%
70%
80%
90%
100%
Methylated sets (Mixture dataset 2)
Nanopolish (r = 0.8405 , r2 = 0.7065 , RMSE = 0.1802)
DeepSignal (r = 0.9344 , r2 = 0.8731 , RMSE = 0.1149)
Megalodon (r = 0.9822 , r2 = 0.9647 , RMSE = 0.0638)
Tombo (r = 0.8467 , r2 = 0.7170 , RMSE = 0.1707)
Guppy (r = 0.9104 , r2 = 0.8288 , RMSE = 0.1515)
METEORE (RF) (r = 0.9842 , r2 = 0.9687 , RMSE = 0.0618)
METEORE (REG) (r = 0.9842 , r2 = 0.9687 , RMSE = 0.0589)

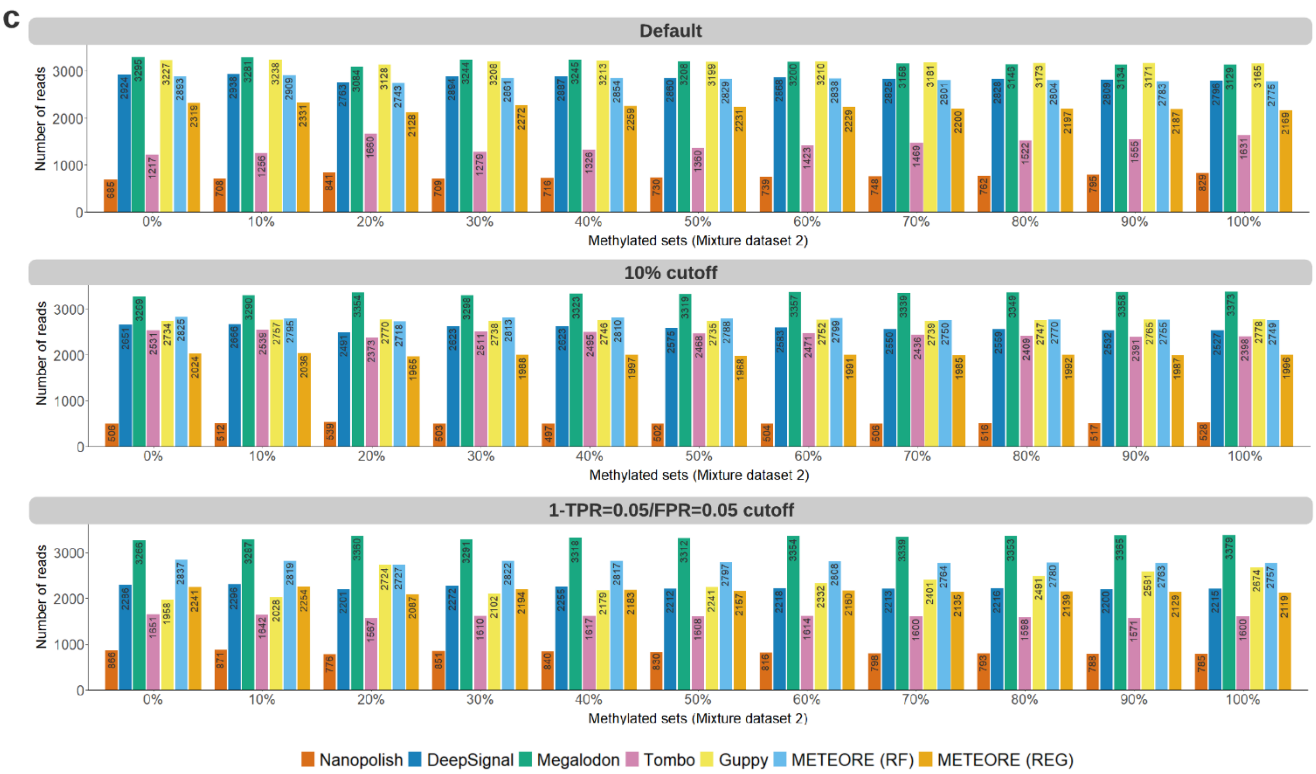

Supplementary Figure 6. Accuracy analysis using a double cutoff and discarding reads. (a) Violin plots showing the predicted methylation frequencies (y axis) for each control mixture set with a given proportion of methylated reads (x axis) from the mixture dataset 2 for the five tested tools plus METEORE combining Megalodon and DeepSignal using random forest (RF) and regression (REG) models, after discarding 10% of the reads with a score closest to the value corresponding to the intersection between FPR and 1-TPR. The Pearson's correlation (r), coefficient of determination (r^2) and the root mean square error (RMSE) are given for each tool. **(b)** Similar plot as **(a)** but considering the scores at which FPR=0.05 and 1-TPR=0.05 and removing all sites in reads with a score between these two values. Cutoffs are given in Supplementary Table 4. **(c)** Total number of reads reported by each method in different approaches: default setting of each method (top), the use of a double cutoff to remove 20% of the reads (middle) and the use of a double cutoff to remove reads with the scores between the cutoff values at FPR=0.05 and 1-TPR=0.05 (bottom). The number of reads are shown inside each bar.

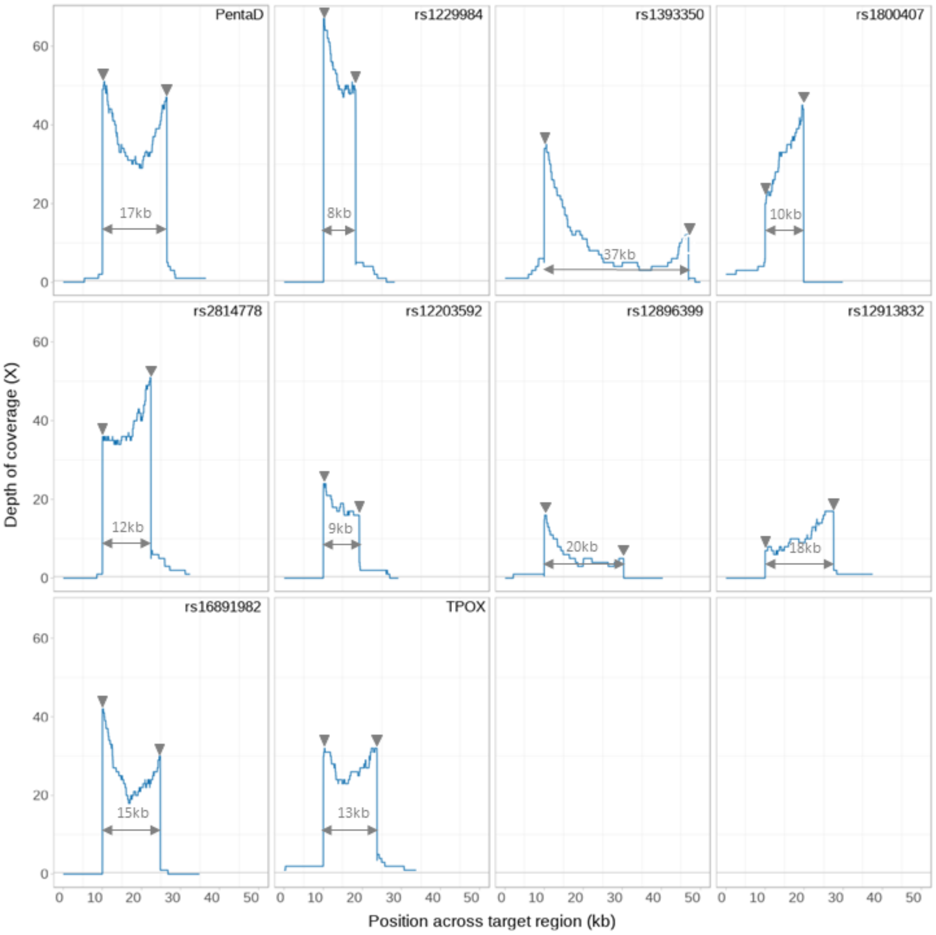

Supplementary Figure 7. Coverage plots for the 10 regions targeted with the nCATS protocol. The target locus is shown in the top right corner of each panel. Details of these ten forensically relevant regions used for the nCATS protocol are given in Supplementary Table 5. For each of our 10 sequenced regions, we show the number of reads (y axis) aligning at each position along the region (x axis). The boundaries and length of each region are also indicated. For the coverage, reads mapped in forward and reverse were considered.

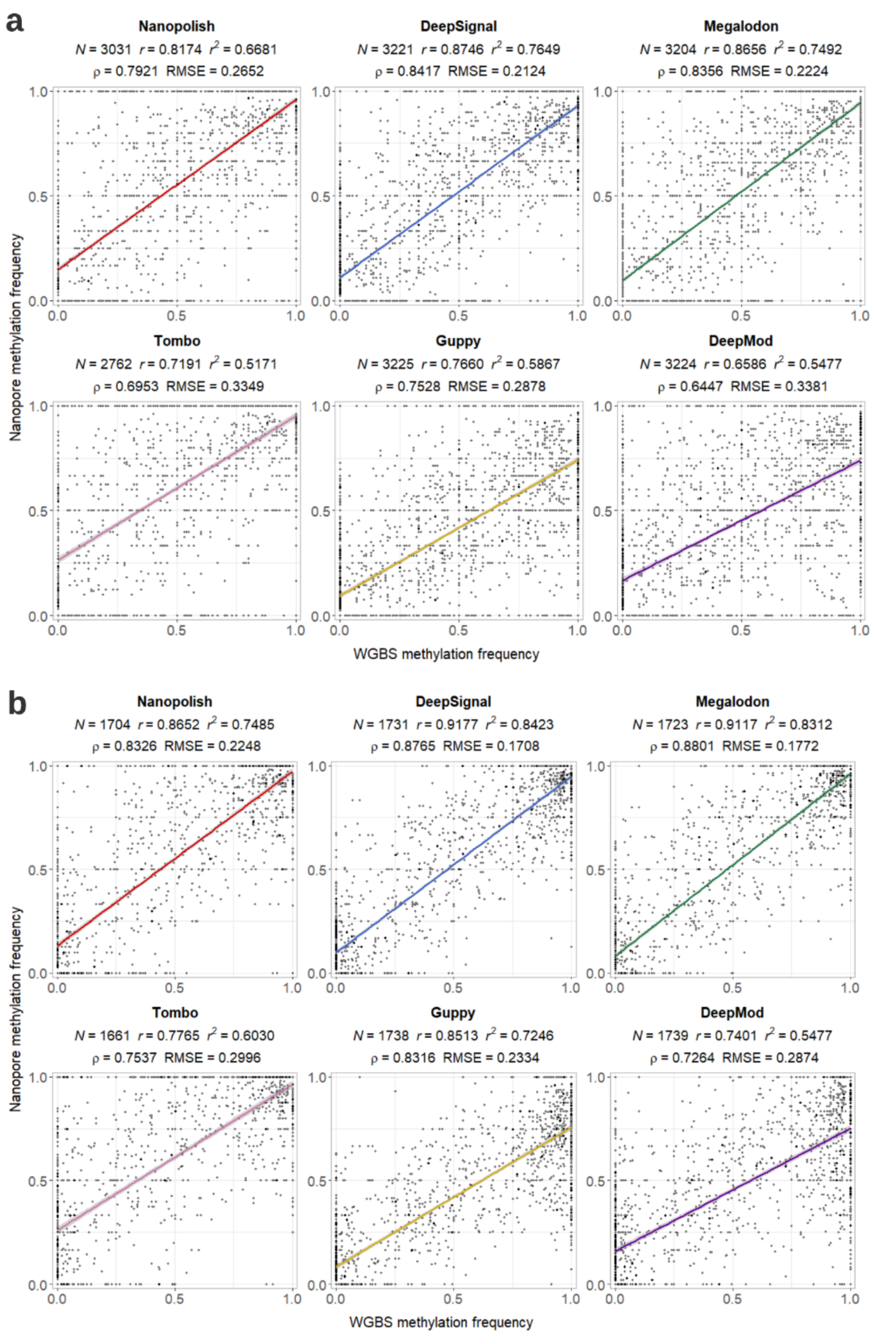

Supplementary Figure 8. Comparison of the nCATS data with whole genome bisulfite sequencing (WGBS). For each tool, we show the methylation fraction predicted by each tool (y axis) and the fraction calculated from WGBS (x axis), using either **(a)** individual predictions on both strands or **(b)** combined predictions from both strands. The number of sites (N), the Pearson's correlation (r), coefficient of determination (r^2), the Spearman's rank correlation (ρ), and the root mean square error (RMSE) are provided for each tested tool. The plots include the correlation bands.

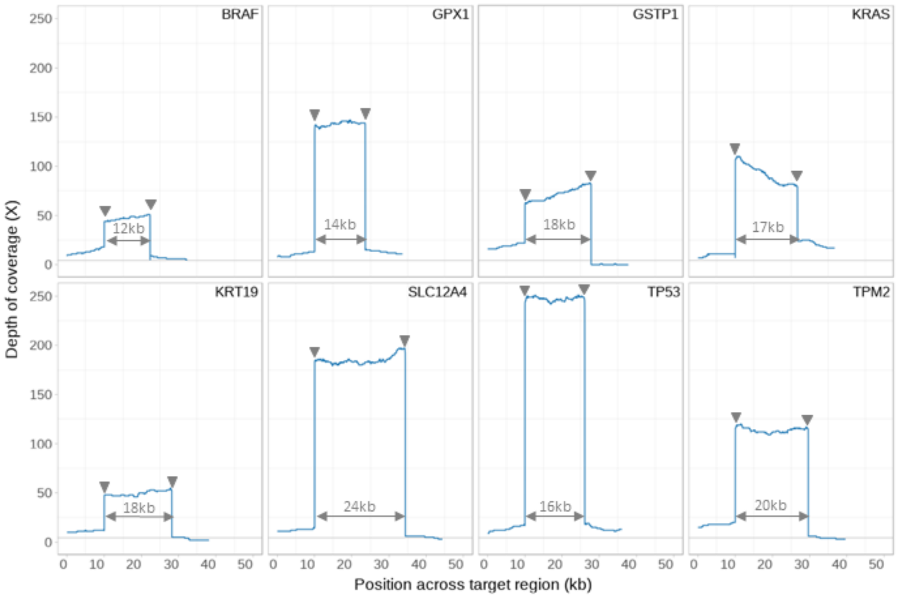

Supplementary Figure 9. Coverage plots for the regions targeted with the nCATS protocol from Gilpatrick et al. 2020. For each of the 8 regions tested in Gilpatrick et al. (2020), we show the number of reads (y axis) aligning at each position along the region (x axis). The boundaries and length of each region are also indicated. For the coverage, reads mapped in forward and reverse were considered.

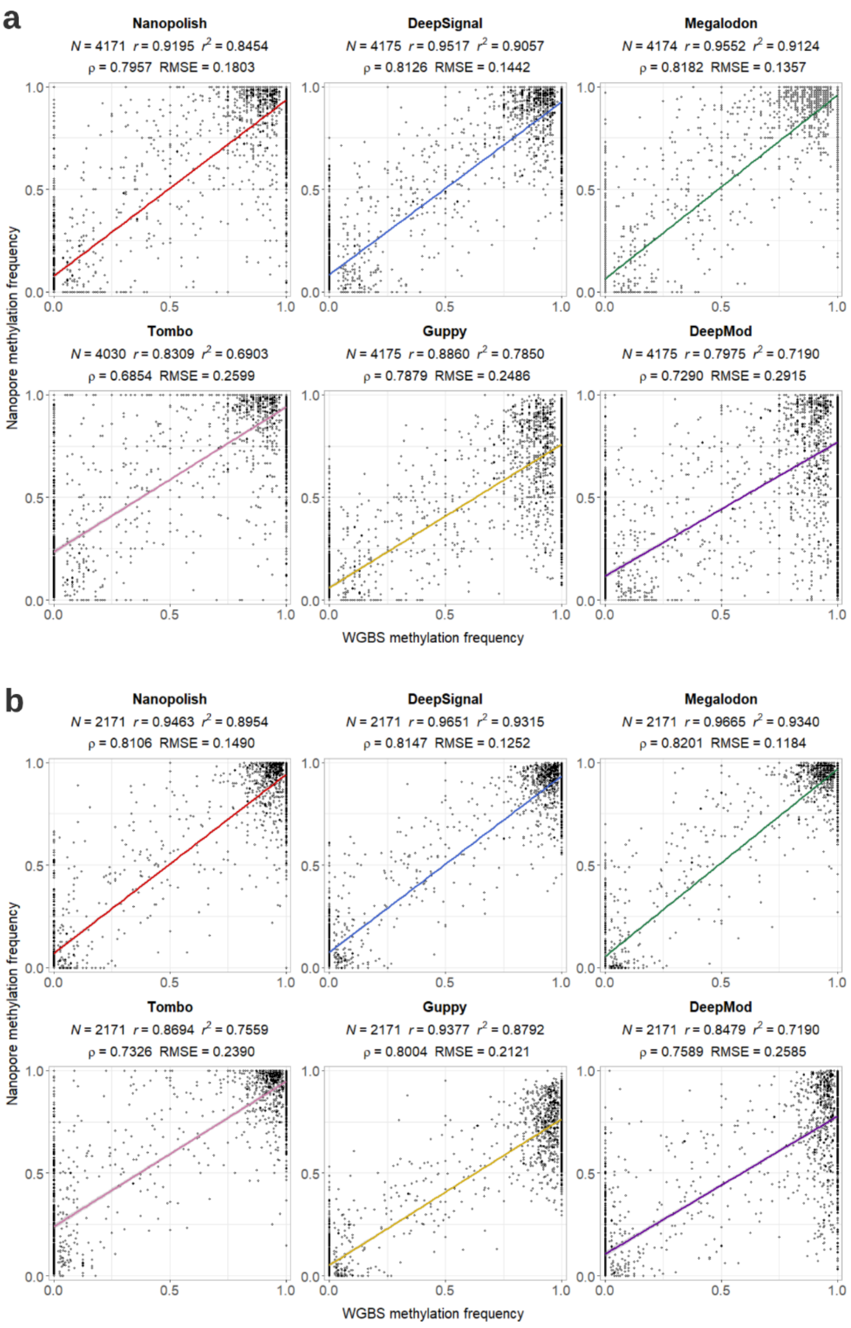

Supplementary Figure 10. Comparison of the nCATS data from Gilpatrick et al. with whole genome bisulfite sequencing (WGBS) data. For each tool, we show the methylation fraction predicted by each tool (y axis) and the fraction calculated from WGBS (x axis), using either **(a)** individual predictions on both strands or **(b)** combined predictions from both strands. The number of sites (N), the Pearson's correlation (r), coefficient of determination (r^2), the Spearman's rank correlation (ρ), and the root mean square error (RMSE) are provided for each tested tool. The plots include the correlation bands

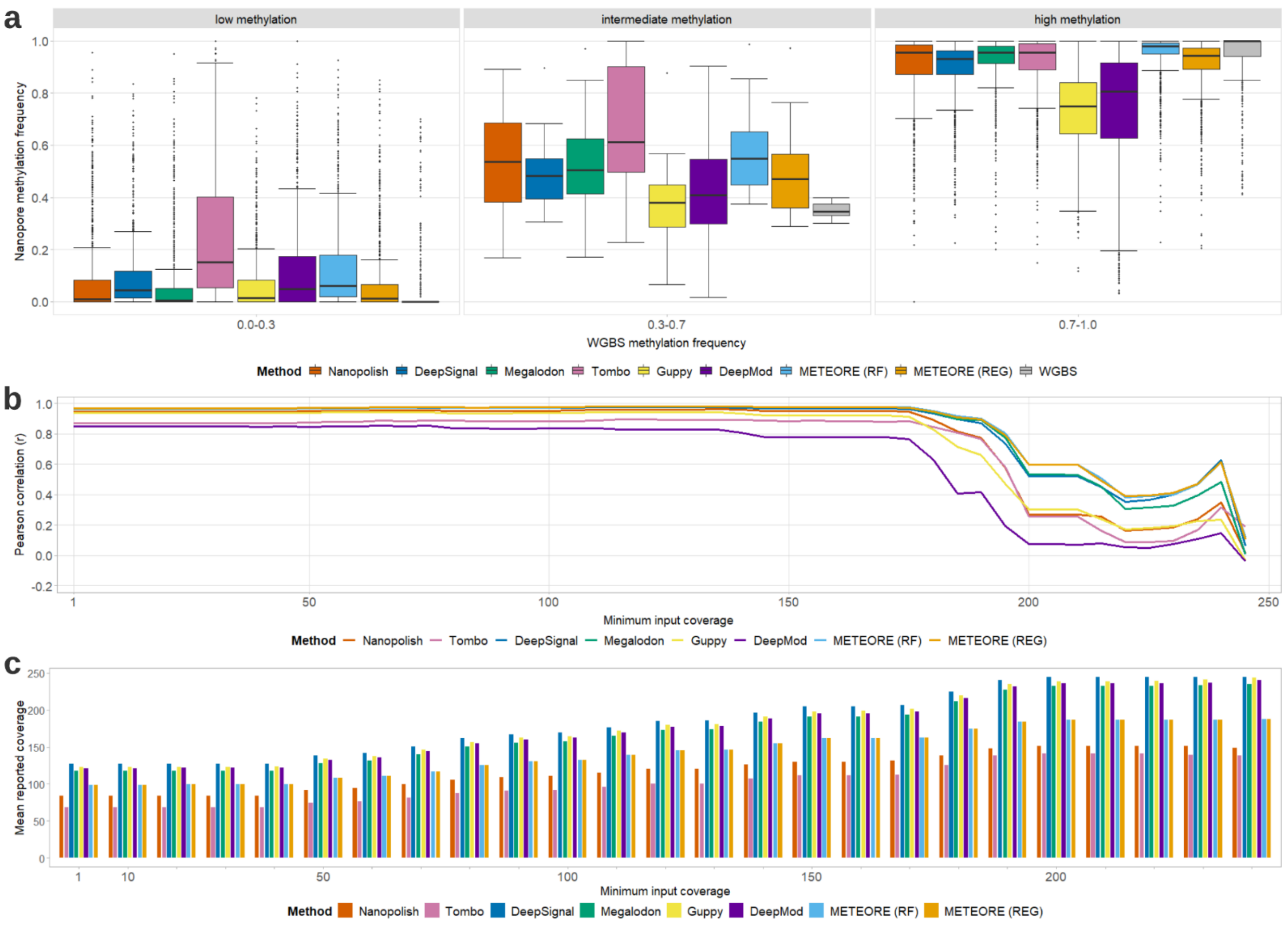

Supplementary Figure 11. Comparison of CpG methylation frequencies from whole genome bisulfite sequencing (WGBS) Illumina data with Cas9-targeted Nanopore data from Gilpatrick et al. (a) Distribution of Nanopore methylation calls (n = 2171) across three WGBS methylation bins unmethylated or lowly methylated (0.0-0.3) (n = 849), intermediate methylation (0.3-0.7) (n = 97), and highly or fully methylated (0.7-1.0) (n = 1225). We show the seven tested tools: Nanopolish, DeepSignal, Megalodon, Tombo, Guppy, DeepMod, and METEORE. For METEORE, we used the combination of Megalodon and DeepSignal using either a random forest model (RF) or a regression model (REG). In the boxplots, the lower and upper boundaries of the box are the first and third quartiles of the data, respectively, with the median indicated by a thick black line. The lower and upper whiskers extend to 1.5 times the interquartile range. The outliers are represented by the black dots. **(b)** Pearson's correlation (r) (y axis) between methylation frequencies calculated from Nanopore by each of the tested tools and WGBS at sites with predictions from both strands combined at each level of minimal input coverage (x axis), i.e., minimum number of Nanopore reads considered per site as reported from the BAM file. **(c)** Mean reported coverage (y axis), using the coverage reported by each tool for each site, at each value of minimum input coverage in (b) (x axis). METEORE (RF) is the combination of DeepSignal and Megalodon using a random forest (parameters: max_depth=3 and n_estimator=10). METEORE (REG) is the combination of DeepSignal and Megalodon using a regression model

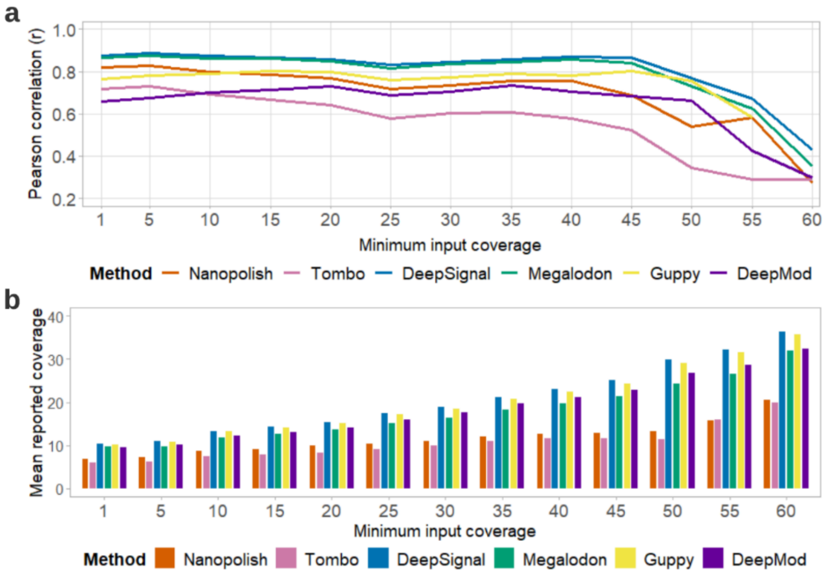

Supplementary Figure 12. Comparison of CpG methylation frequencies from whole genome bisulfite sequencing (WGBS) Illumina data with Cas9-targeted Nanopore data independently on each strand. (a) Pearson's correlation (r) (y axis) between methylation frequencies calculated from Nanopore by each of the tested tools and WGBS at individual sites at each level of minimal input coverage, i.e., minimum number of Nanopore reads considered per site as reported from the BAM file (x axis). **(b)** Mean reported coverage (using the coverage reported by each tool for each site) considered at each value of minimum input coverage in (a). METEORE is not included since it performs predictions only combining both strands.

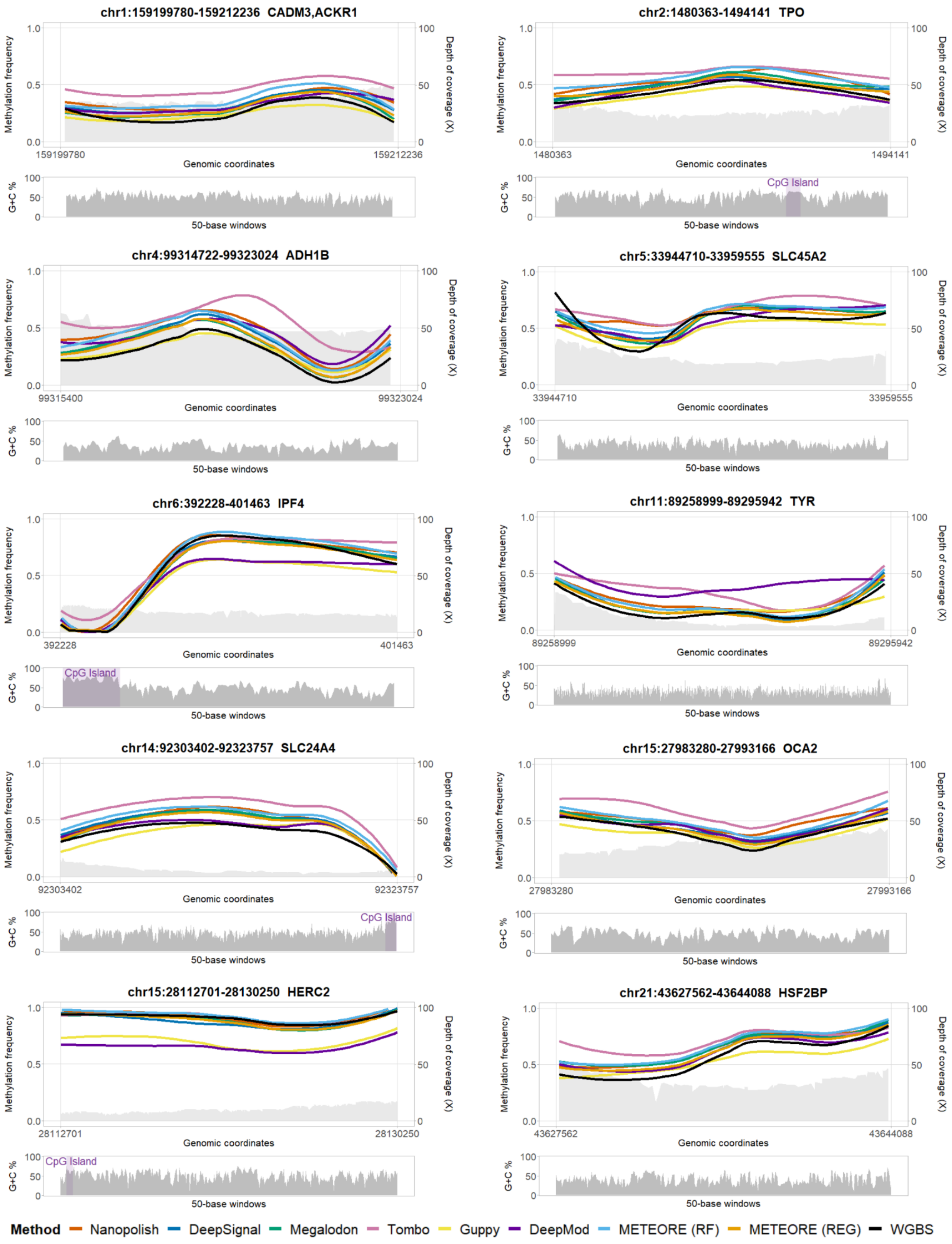

Supplementary Figure 13. Comparison of CpG methylation frequencies from whole genome bisulfite data (WGBS) and Nanopore across our 10 targeted regions. Locally Weighted Scatterplot Smoothing (LOESS) smoothing line plots of methylation calls from WGBS Illumina and Nanopore data detected by the eight tested tools. We show METEORE with the combination of Megalodon and DeepSignal using either a random forest model (RF) or a regression model (REG). The plots include the Nanopore coverage, shown as a light grey area. Below each plot, we include the GC-content of the region.

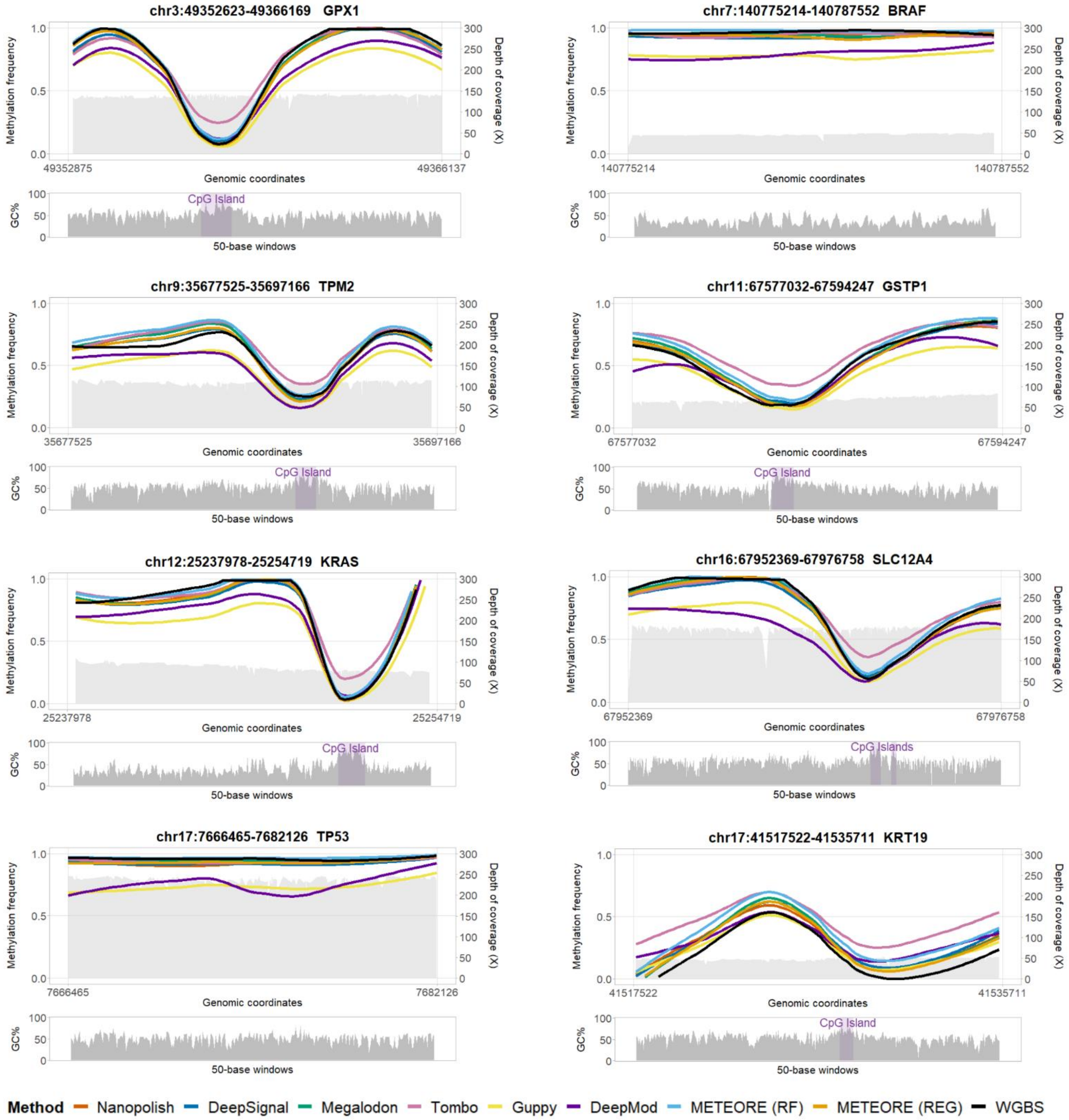

Supplementary Figure 14. Comparison of CpG methylation frequencies from whole genome bisulfite data (WGBS) and Nanopore across the 8 regions tested in Gilpatrick et al. Locally Weighted Scatterplot Smoothing (LOESS) smoothing line plots of methylation calls from WGBS Illumina and Nanopore data detected by the eight tested tools. We show METEORE with the combination of Megalodon and DeepSignal using either a random forest model (RF) or a regression model (REG). The plots include the Nanopore coverage, shown as a light grey area. Below each plot, we include the GC-content of the region.

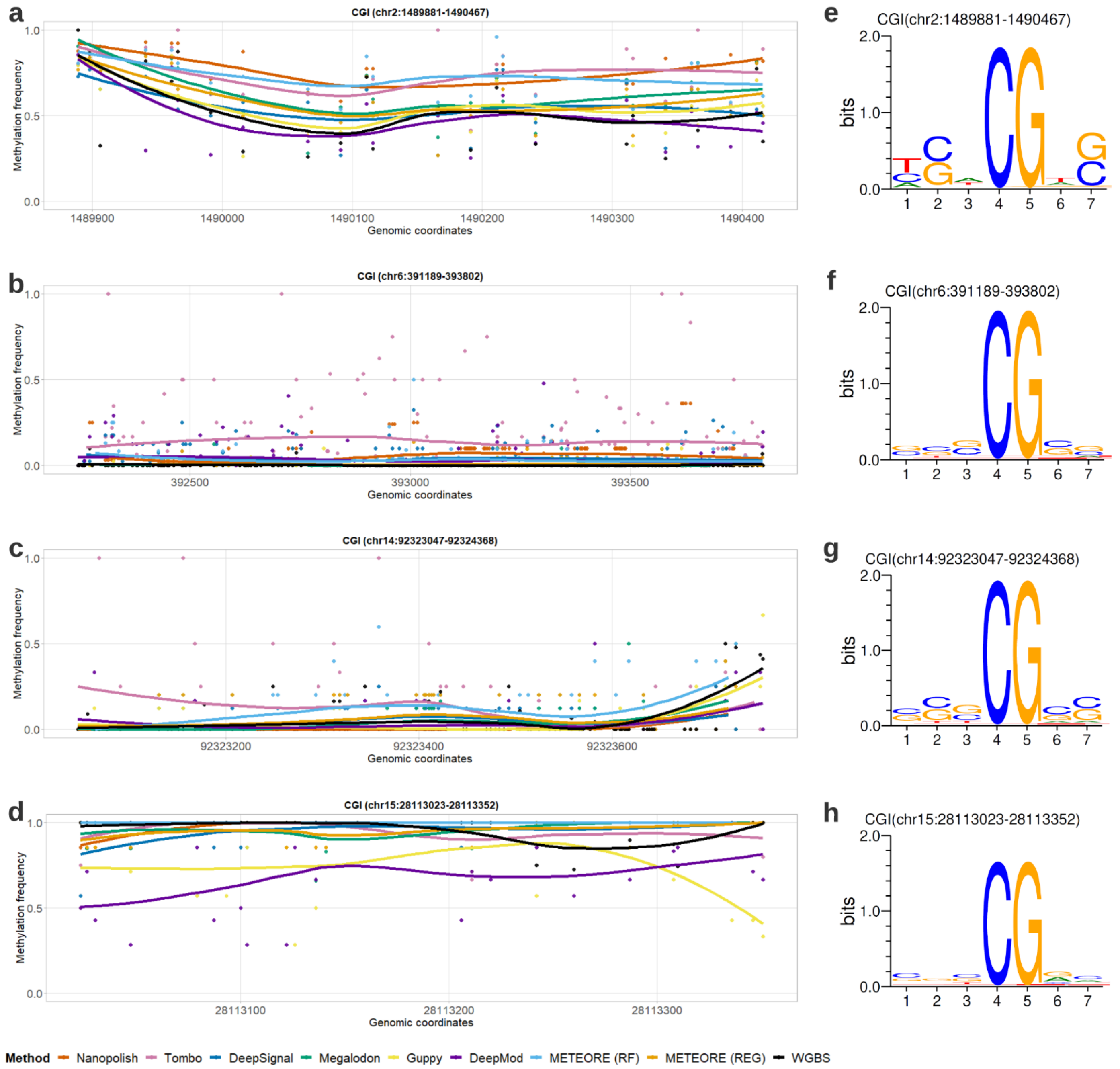

Supplementary Figure 15. Zoom in on the CpG Islands (CGIs) for the comparison of CpG methylation predictions from Nanopore with whole genome bisulfite sequencing (WGBS). (a-d) show a zoom in of the LOESS smoothing line plots of methylation frequency (y axis) with individual methylation calls (points) for nine different methods for the CGIs in four of our ten target regions shown in Supplementary Fig. 13. (**e-h)** show the sequence logos showing information content in bits of the motifs (7-mers) at the CpG sites in the same CGIs shown in (a-d).

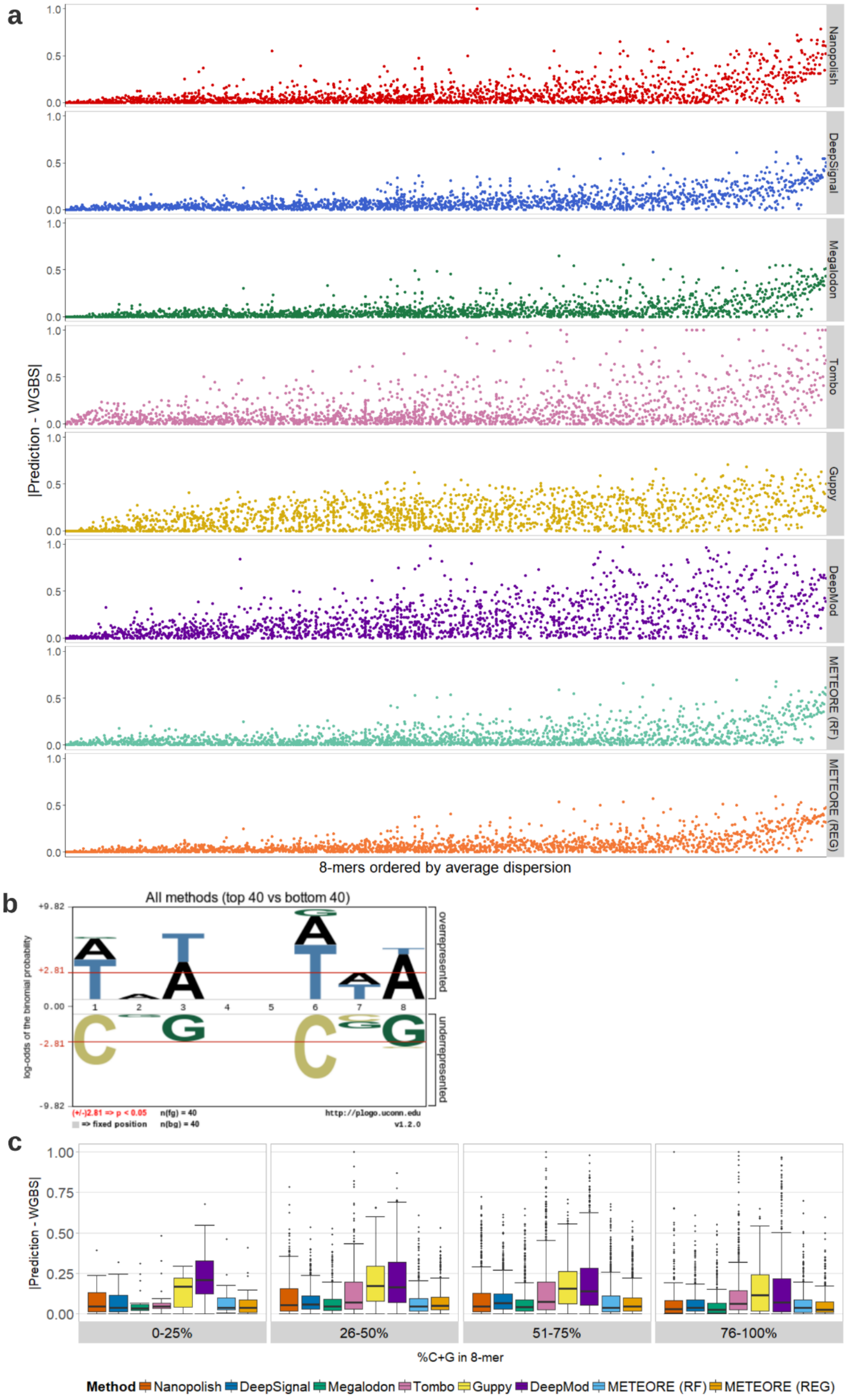

Supplementary Figure 16. Sequence context analysis. (a) Absolute difference between methylation frequencies from Nanopore-based methylation detection tools and whole genome bisulfite sequencing (WGBS) data for all 8-mers with a CpG in the middle (NNNCGNNN) (x axis) ordered from left to right in ascending order according to the average value of the absolute difference (y axis) across all tools. **(b)** Sequence logo generated by the plogo Web tool[27] for the top/bottom 40 8-mers from the ranking in (a). The bottom 40 8-mers were considered the foreground (fg) dataset, as shown in the upper panel, whereas the top 40 8-mers where considered the background (bg) dataset, as shown in the lower panel. pLogo uses binomial test to

determine the statistical significance of the residues in the k-mers. One-tailed significance was determined by computing the area under the desired side (fg and bg datasets) of a probability distribution. Residues are scaled proportional to the log odds of the significance of foreground dataset (overrepresentation) versus the significance of background dataset (underrepresentation) (y axes) and stacked according to the statistical significance, with the most significant residues positioned closest to the x axis. The significance level (α) and the number of foreground and background sequences used, i.e., n(fg) and n(bg) values, are given at the bottom of each logo. The red horizontal lines correspond to α = 0.05 following Bonferroni correction. The following residues from the foreground dataset are considered as statistically significant, i.e. p-value < 0.05, as calculated by pLogo: T at 1st, 3rd and 6th positions (p-values = 0.002, 0.025 and 3.943e-5 respectively) and A at 3rd, 6th and 8th positions (p-values = 0.003, 0.025 and 4.147e-4 respectively) **(c)** Same absolute difference of methylation frequencies in 8-mers (n = 1911) from (a) (y axis) but stratified by the percentage of C and G residues in the 8-mers (0-25%: n = 20, 26-50%: n = 317, 51-75%: n = 1093 and 76-100%: n = 481), separated by tool. In the boxplots, the lower and upper boundaries of the box are the first and third quartiles of the data, respectively, with the median indicated by a thick black line. The lower and upper whiskers extend to 1.5 times the interquartile range. The outliers are represented by the black dots.

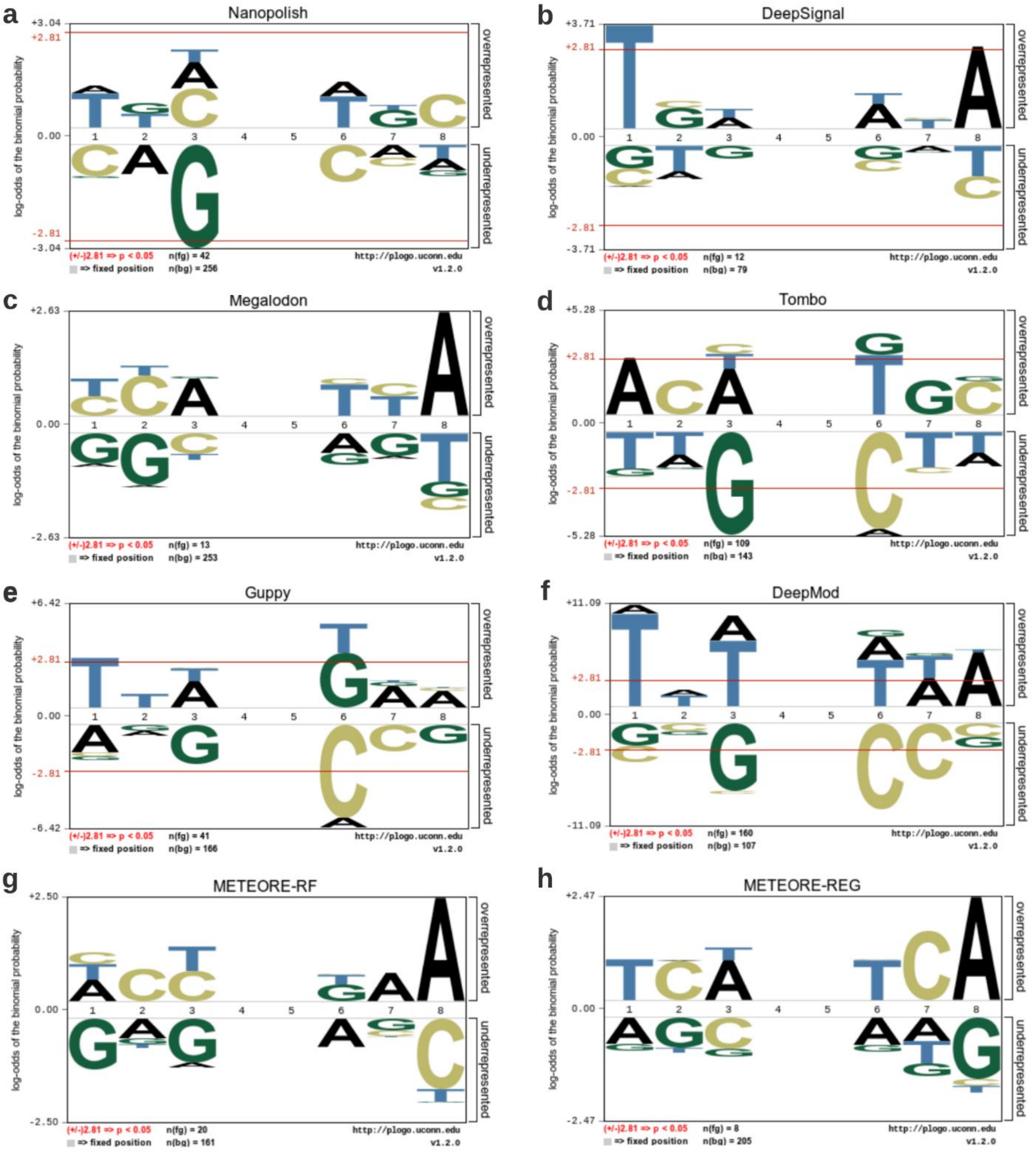

Supplementary Figure 17. Sequence context associated to sites of high and low discrepancy with whole genome bisulfite sequencing (WGBS). Here we show the pLogo plots for **(a)** Nanopolish, **(b)** DeepSignal, **(c)** Megalodon, **(d)** Tombo, **(e)** Guppy, **(f)** DeepMod, **(g)** METEORE with random forest model (RF) and **(h)** METEORE with regression model (REG). For each method, CG-containing 8-mers (NNNCGNNN) were labelled as "low discrepancy" (foreground (fg) dataset, as shown in the upper panel of each plot) if the discrepancy with WGBS (in absolute value) was >0.5, or as "high discrepancy" (background (bg) dataset, as shown in the lower panel of each plot) if the discrepancy with WGBS was exactly 0. The pLogo Web tool[27] uses binomial test to determine the statistical significance of the residues in the k-mers. One-tailed significance was determined by computing the area under the desired side (foreground and background datasets) of a probability distribution. Residues are scaled proportional to the log odds of the significance of foreground dataset (overrepresentation) versus the significance of background dataset (underrepresentation) (y axes) and stacked according to the statistical significance, with the most significant residues positioned closest to the x axis. The significance level (α) and the number of foreground and background sequences used, i.e., n(fg) and n(bg) values, are given at the bottom of each logo. The red horizontal lines correspond to α = 0.05 following Bonferroni correction. The following residues from the foreground dataset are considered as statistically

significant, i.e. p-value < 0.05, as calculated by pLogo: T at 1st position (p-value = 0.006) and A at 8th position (p-value = 0.035) for DeepSignal; T at 1st position (p-value = 0.024) and G at 6th position (p-value = 0.013) for Guppy; T at 1st, 3rd and 6th position (p-values = 2.484e-9, 2.072e-6 and 2.540e-4 respectively) and A at 7th and 8th position (p-values = 0.026 and 4.110e-5 respectively) for DeepMod.

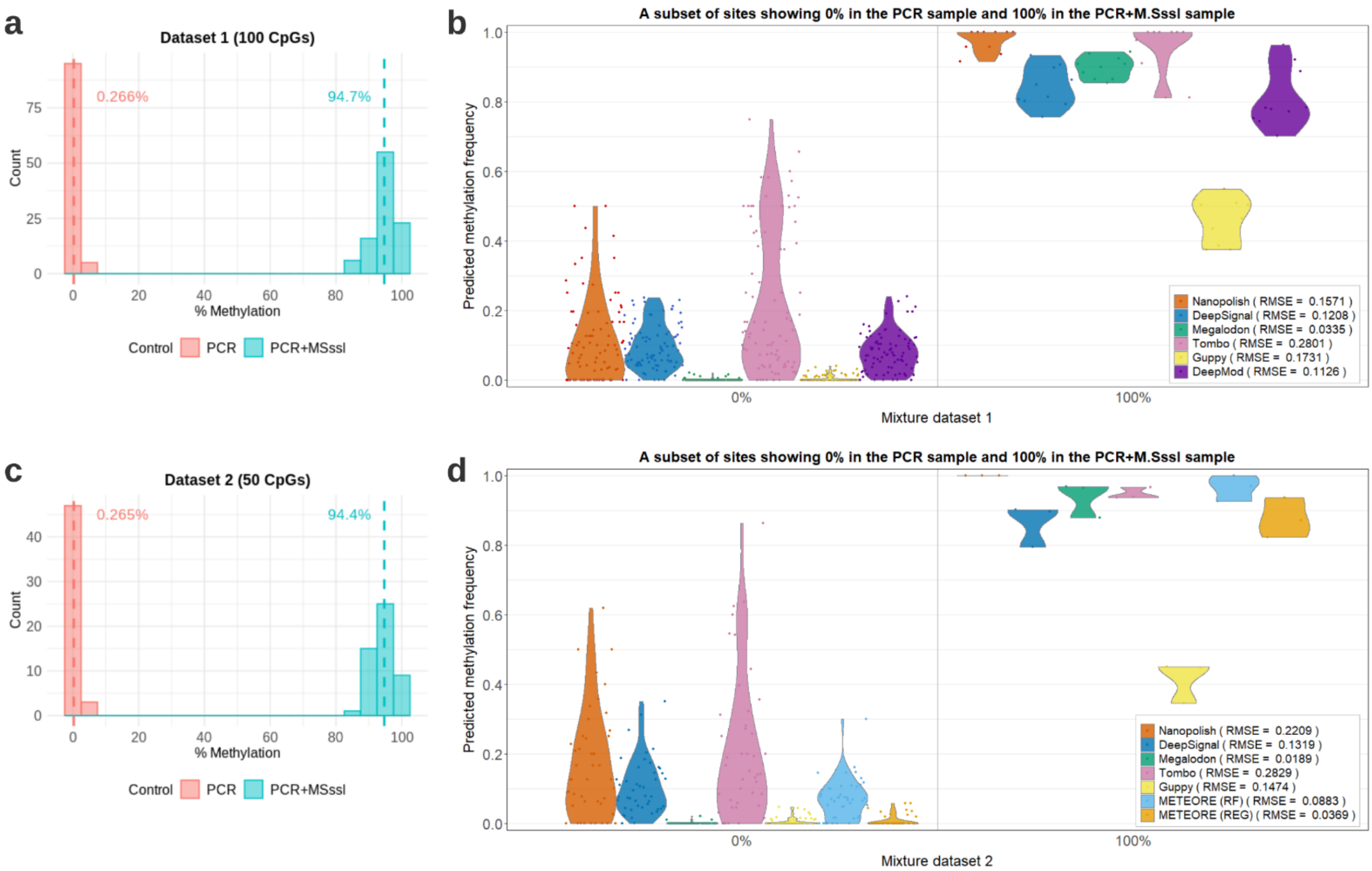

Supplementary Figure 18. Confirmation of the methylation levels of control samples using whole genome bisulfite sequencing (WGBS) data. (a) Distribution plot showing the methylation percentage for the 100 target sites in the mixture dataset 1 in the positive (PCR+M.SssI) (mean methylation = 94.7%) and negative (PCR) (mean methylation = 0.266%) controls. **(b)** Violin plots showing the distribution of predicted methylation frequencies (y axis) in the sites from dataset 1 that in (a) showed exactly 0% methylation in the negative control and exactly 100% methylation in the positive control, using WGBS. The root mean square error (RMSE) is given for each tool. **(c)** Distribution plot showing methylation percentage for the 50 target sites in the mixture dataset 2 in the positive (mean = 94.4%) and negative (mean = 0.265%) controls. **(d)** Violin plots showing the distribution of predicted methylation frequencies (y axis) in the sites from the mixture dataset 2 that in (c) showed exactly 0% methylation in the negative control and exactly 100% methylation in the positive control, using WGBS. The root mean square error (RMSE) is given for each tool.

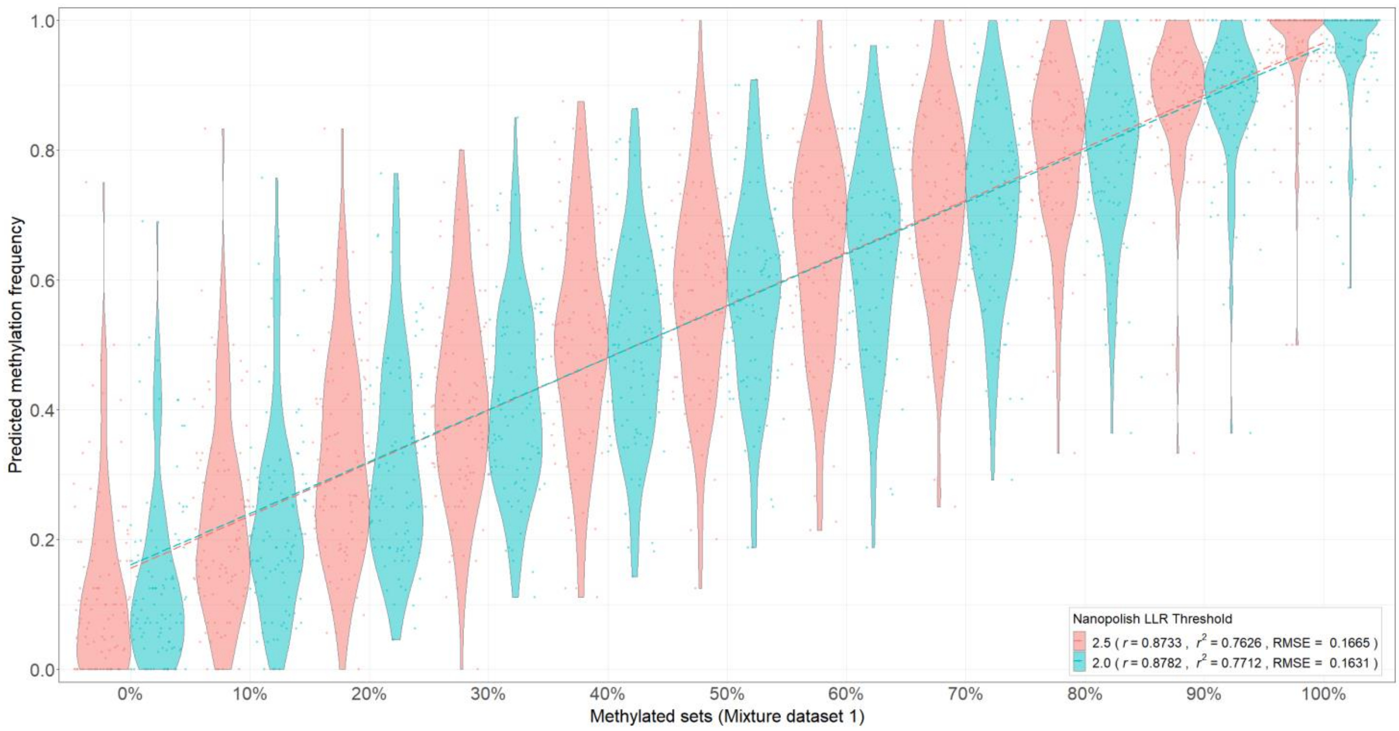

Supplementary Figure 19. Comparison of methylation frequencies using log-likelihood ratio (LLR) thresholds of 2.5 and 2.0 for Nanopolish. Two different LLR thresholds were used to make a per-site methylation call on control mixture dataset 1.

a

Set name	% methylated	Total reads	Unmethylated reads (PCR)	Methylated reads (PCR+M.Sssl)
m0	0	2390	2390	0
m10	10	2437	2182	255
m20	20	2431	1946	485
m30	30	2434	1714	720
m40	40	2410	1458	952
m50	50	2432	1231	1201
m60	60	2414	981	1433
m70	70	2420	739	1681
m80	80	2410	498	1912
m90	90	2399	256	2143
m100	100	2225	0	2225

b

Set name	% methylated	Total reads	Unmethylated reads (PCR)	Methylated reads (PCR+M.Sssl)
m0	0	3420	3420	0
m10	10	3426	3081	345
m20	20	3413	2745	668
m30	30	3423	2410	1013
m40	40	3415	2068	1347
m50	50	3434	1736	1698
m60	60	3403	1377	2026
m70	70	3406	1039	2367
m80	80	3398	690	2708
m90	90	3396	354	3042
m100	100	3383	0	3383

Supplementary Table 1. Methylation control mixtures. We describe the mixture dataset 1 **(a)** and 2 **(b)** used for the benchmarking of different methylation proportions built from fully unmethylated and fully methylated reads.

Unmethylated if freq < 0.1, methylated if freq > 0.9					
	Accuracy	Specificity	Precision	Recall	Error rate
**Nanopolish**	0.675	0.490	0.628	0.860	0.325
**DeepSignal**	0.410	0.590	0.359	0.230	0.590
**Megalodon**	0.825	1.000	1.000	0.650	0.175
**Tombo**	0.588	0.384	0.564	0.790	0.412
**Guppy**	0.500	1.000	NA	0.000	0.500
**DeepMod**	0.495	0.680	0.492	0.310	0.505

Unmethylated if freq < 0.2, methylated if freq > 0.8					
	Accuracy	Specificity	Precision	Recall	Error rate
**Nanopolish**	0.860	0.800	0.821	0.920	0.140
**DeepSignal**	0.805	0.910	0.886	0.700	0.195
**Megalodon**	0.970	1.000	1.000	0.940	0.030
**Tombo**	0.794	0.616	0.719	0.970	0.206
**Guppy**	0.500	1.000	NA	0.000	0.500
**DeepMod**	0.790	0.970	0.953	0.610	0.210

Unmethylated if freq < 0.3, methylated if freq > 0.7					
	Accuracy	Specificity	Precision	Recall	Error rate
**Nanopolish**	0.935	0.900	0.907	0.970	0.065
**DeepSignal**	0.930	1.000	1.000	0.860	0.070
**Megalodon**	0.980	1.000	1.000	0.960	0.020
**Tombo**	0.839	0.697	0.766	0.980	0.161
**Guppy**	0.505	1.000	1.000	0.010	0.495
**DeepMod**	0.935	1.000	1.000	0.870	0.065

Unmethylated if freq < 0.4, methylated if freq > 0.6					
	Accuracy	Specificity	Precision	Recall	Error rate
**Nanopolish**	0.955	0.930	0.933	0.980	0.045
**DeepSignal**	0.975	1.000	1.000	0.950	0.025
**Megalodon**	1.000	1.000	1.000	1.000	0.000
**Tombo**	0.884	0.778	0.818	0.990	0.116
**Guppy**	0.600	1.000	1.000	0.200	0.400
**DeepMod**	0.975	1.000	1.000	0.950	0.025

Unmethylated if freq < 0.5, methylated if freq > 0.5					
	Accuracy	Specificity	Precision	Recall	Error rate
**Nanopolish**	0.975	0.970	0.970	0.980	0.025
**DeepSignal**	0.990	1.000	1.000	0.980	0.010
**Megalodon**	1.000	1.000	1.000	1.000	0.000
**Tombo**	0.915	0.838	0.861	0.990	0.085
**Guppy**	0.720	1.000	1.000	0.440	0.280
**DeepMod**	0.980	1.000	1.000	0.960	0.020

Supplementary Table 2. Per-site performance. The table shows the accuracies in fully methylated or fully unmethylated CpG sites for the six tested tools using two different methylation frequency thresholds to classify methylated and unmethylated sites. For each pair of thresholds (a,b), we defined a site to be unmethylated if the predicted methylation frequency was <a, and methylated if the predicted methylation frequency was >b, for (a,b) = (0.1,0.9), (0.2,0.8), (0.3,0.7), (0.4,0.6), and (0.5,0.5).

	Nanopolish	DeepSignal	Megalodon	Tombo	Guppy	METEORE (RF)	METEORE (REG)
Maximum of (TPR –FPR)	Cutoff= 1.03<br>TPR =0.87<br>FPR=0.15	Cutoff= -0.05<br>TPR =0.86<br>FPR=0.10	Cutoff= -0.98<br>TPR =0.91<br>FPR=0.04	Cutoff= -0.13<br>TPR =0.83<br>FPR=0.18	Cutoff= -1.35<br>TPR =0.69<br>FPR=0.05	Cutoff= 0.62<br>TPR =0.94<br>FPR=0.03	Cutoff= 0.33<br>TPR =0.95<br>FPR=0.03
Minimum of $(FPR\text{-}0)^2$ + $(TPR\text{-}1)^2$	Cutoff= 1.04<br>TPR=0.87<br>FPR=0.15	Cutoff= -0.19<br>TPR =0.87<br>FPR=0.11	Cutoff= -1.36<br>TPR =0.92<br>FPR=0.05	Cutoff= -0.11<br>TPR =0.82<br>FPR=0.18	Cutoff= -1.70<br>TPR =0.73<br>FPR=0.12	Cutoff= 0.55<br>TPR =0.95<br>FPR=0.04	Cutoff= 0.33<br>TPR =0.96<br>FPR=0.04

Supplementary Table 3. Single score cutoffs. Cutoffs obtained by maximising the value of TPR-FPR (first row) or by minimizing the value of $(FPR\text{-}0)^2$ + $(TPR\text{-}1)^2$ (second row). In both optimization we used all reads from the mixture dataset 1. These cutoffs were applied to the per-read data generated by each tool. For all these tools except for Tombo, if a read with a score above the cutoff, we consider it as methylated, and unmethylated for the scores below the cutoff. For Tombo, a read is considered methylated if its score is below the cutoff, and unmethylated for a score above the cutoff. All values were rounded up to 2 decimal places. For METEORE REG, both strategies led to exactly the same cutoffs after rounding up.

	Nanopolish	DeepSignal	Megalodon	Tombo	Guppy	METEORE (RF)	METEORE (REG)
Remove **10%** of reads around the cross point of FPR and 1-TPR curves	(-3.58 ,5.80)	(-0.60, 0.09)	(-2.07, -1.20)	(-0.54,0.34)	(-1.93, -0.91)	(0.47,0.56)	(0.20,0.46)
Remove the reads that fall between the score at **1-TPR=0.05** and **FPR=0.05**	(-0.65, 3.53)	(-1.07, 0.60)	(-2.14, -1.29)	(-1.82, 1.66)	(-2.41, -1.31)	(0.48,0.55)	(0.29, 0.34)

Supplementary Table 4. Double score cutoffs. Cutoffs obtained by removing 10% of reads around the intersection point of the FPR curve and 1-TPR curve (first row) or removing the cases that fall between the score at 1- TPR = 0.05 and FPR = 0.05 (second row). In the first optimization we used all reads from the mixture dataset 1. In the second optimization we used the fully methylated and fully unmethylated sets from mixture dataset 1. For each double cutoff (a,b), all sites in reads with score < a are considered unmethylated, with score > b are considered methylated, and all cases between these values are discarded. For Tombo the score scale has the opposite orientation, i.e., a read is considered methylated if its score is < a, and unmethylated for a score > b. METEORE (RF) is the combination of DeepSignal and Megalodon using a random forest (parameters: max_depth=3 and n_estimator=10). METEORE (REG) is the combination of DeepSignal and Megalodon using a regression model. In the REG model, filtering is symmetric by the ranking of scores about the tipping point, i.e., 5% of reads with scores lower than the tipping point and 5% of reads with scores higher than the tipping point.

Chromosome	Start position	End position	Size (nt)	Target locus	Type of variants	Associated gene(s)
chr1	159199780	159212236	12456	rs2814778	aiSNP	CADM3, ACKR1
chr2	1480363	1494141	13778	TPOX	STR	TPO
chr4	99314722	99323024	8302	rs1229984	aiSNP	ADH1B
chr5	33944710	33959555	14845	rs16891982	piSNP	SLC45A2
chr6	392228	401463	9235	rs12203592	piSNP	IPF4
chr11	89258999	89295942	36943	rs1393350	piSNP	TYR
chr14	92303402	92323757	20355	rs12896399	piSNP	SLC24A4
chr15	27983280	27993166	9886	rs1800407	piSNP	OCA2
chr15	28112701	28130250	17549	rs12913832	piSNP	HERC2
chr21	43627562	43644088	16526	PentaD	STR	HSF2BP

Supplementary Table 5. Ten forensically relevant regions used for the nCATS protocol. The table provides the coordinates (GRCh38) of the ten regions used to sequence native DNA with the nCATS protocol. The table also indicates whether the region contains an ancestry-informative SNP (aiSNP), a phenotypic-informative SNP (piSNP), or a short tandem repeat (STR).

Target	Guide RNA sequence	PAM	Cleaved site
rs12913832	CTTGTTCTCAATCCAACGAG	CGG	chr15:28112701(+)
	GATCAGATGACCATGTTCGA	AGG	chr15:28130250(-)
rs1800407	GTAGAGCTCTAACTAAGTGG	AGG	chr15:27983280(+)
	TATCCAATCCTGCTGACCAG	TGG	chr15:27993166(-)
rs12896399	GCTGGAACGCCCCATCAACA	CGG	chr14:92303402(+)
	GAGTGCAATCAGTGGCCGAG	CGG	chr14:92323757(-)
rs16891982	TGTGATCACCACGACGACAA	CGG	chr5:33944710(+)
	GAGTGCAACGAGGAACTAAG	AGG	chr5:33959555(-)
rs1393350	TCCTTGCTGCACGAATCAGT	GGG	ch11:89258999(+)
	GCTGGATGTGTTATAGACGC	TGG	chr11:89295942(-)
rs12203592	TAAGGGGCCCAAGCTCACGG	CGG	chr6:392228(+)
	ACGTGGTCAGCTCCTTCACG	AGG	chr6:401463(-)
TPOX	CGTATTTGAAAGATCCACGG	TGG	chr2:1480363(+)
	CTTACGTAAGAGTTGAATGG	TGG	chr2:1494141(-)
Penta D	CGGTACCTATCCCAGAACTA	TGG	chr21:43627562(+)
	TAACACGTAGATCATTCACT	TGG	chr21:43644088(-)
rs2814778	CCTACCACGCCATCATCGGT	GGG	chr1:159199780(+)
	GCAATTGTCTTTCAGTGCGT	TGG	chr1:159212236(-)
rs1229984	ACCATCTGCTAACACGTATG	AGG	chr4:99314772(+)
	GCGTTAACATATCTCCACAA	GGG	chr4:99323024(-)

Supplementary Table 6. Guide RNA (gRNA) panel used for the nCATs protocol. The table describe the ten pairs of gRNAs used to target the ten regions from Supplementary Table 3. To enrich for each target region, two gRNAs were used to make a cut on each side, one upstream of the region of interest targeting the positive strand, and the other one downstream targeting the negative strand.

	N	*r*	*r²*	*ρ*	*RMSE*
Nanopolish	2171	0.9463	0.8954	0.8106	0.1490
DeepSignal	2171	0.9651	0.9315	0.8147	0.1252
Megalodon	2171	0.9665	0.9340	0.8201	0.1184
Tombo	2171	0.8694	0.7559	0.7326	0.2390
Guppy	2171	0.9377	0.8792	0.8004	0.2121
DeepMod	2171	0.8479	0.7190	0.7589	0.2585
METEORE (RF)	2171	0.9641	0.9294	0.8259	0.1307
METEORE (REG)	2171	0.9689	0.9387	0.8253	0.1155

Supplementary Table 7. Comparison of CpG methylation frequencies from whole genome bisulfite sequencing (WGBS) Illumina data with Cas9-targeted Nanopore data from Gilpatrick et al. 2020. For each tool we provide the number of sites (N), the Pearson's correlation (r), coefficient of determination (r^2), the Spearman's rank correlation (ρ), and the root mean square error (RMSE) for the comparison of the percentage methylation predicted from Nanopore with the percentage methylation calculated from WGBS data. We show the results for five tested tools and METEORE combining DeepSignal and Megalodon using a random forest (RF) (parameters: max_depth=3 and n_estimator=10) or a regression (REG) model.

	5x	10x	20x	50x
Nanopolish	0.7456	0.7994	0.8095	0.8145
DeepSignal	0.8065	0.8361	0.8438	0.8458
Megalodon	0.8247	0.8400	0.8514	0.8513
Guppy	0.7021	0.7469	0.7595	0.7696
Tombo	0.6245	0.6934	0.7105	0.7142
METEORE (RF)	0.8335	0.8378	0.8511	0.8509
METEORE (REG)	0.8326	0.8461	0.8551	0.8565

Supplementary Table 8. Pearson correlations (r) of methylation frequencies obtained by different Nanopore methylation tools and whole genome bisulfite sequencing (WGBS) at different coverage levels. We subsampled 5x, 10x, 20x, 50x read coverage for 941 CpG sites, using Cas9-targeted Nanopore sequencing data from Gilpatrick et al. 2020.

	N	*r*	r^2	*ρ*	*RMSE*
Nanopolish	1724	0.8648	0.7478	0.8362	0.2171
DeepSignal	1731	0.9196	0.8456	0.8785	0.1693
Megalodon	1723	0.9040	0.8172	0.8753	0.1938
Tombo	1734	0.8037	0.6460	0.7871	0.2551
Guppy	1738	0.7706	0.5938	0.7741	0.2978
METEORE (RF)	1723	0.9217	0.8496	0.8878	0.1736
METEORE (REG)	1723	0.9164	0.8397	0.8871	0.1829

Supplementary Table 9. Comparison of CpG methylation frequencies from whole genome bisulfite sequencing (WGBS) Illumina data with Cas9-targeted Nanopore data for each method using single score thresholds obtained by the maximum value of (TPR-FPR). We used the score cutoffs that maximized TPR-FPR in the mixture dataset 1 (Supplementary Table 3). For each method we provide the number of sites (N), the Pearson's correlation (r), coefficient of determination (r^2), the Spearman's rank correlation (ρ), and the root mean square error (RMSE) for the comparison of the percentage methylation predicted from Nanopore with the percentage methylation calculated from whole genome bisulfite sequencing (WGBS) data. We show the results for five tested tools and METEORE combining DeepSignal and Megalodon using a random forest (RF) (parameters: max_depth=3 and n_estimator=10) or a regression (REG) model.

	N	*r*	r^2	*ρ*	*RMSE*
Nanopolish	2171	0.9265	0.8584	0.7943	0.2061
DeepSignal	2171	0.9654	0.9319	0.8149	0.1246
Megalodon	2171	0.9602	0.9219	0.8195	0.1372
Tombo	2171	0.8936	0.7986	0.7802	0.2343
Guppy	2171	0.9034	0.8162	0.7957	0.2148
METEORE (RF)	2171	0.9668	0.9348	0.8262	0.1221
METEORE (REG)	2171	0.9646	0.9305	0.8263	0.1271

Supplementary Table 10. Comparison of CpG methylation frequencies from whole genome bisulfite sequencing (WGBS) Illumina data with Cas9-targeted Nanopore data from Gilpatrick et al. 2020 for each method using single score thresholds obtained by the maximum value of (TPR-FPR). We used the score cutoffs that maximized TPR-FPR in the mixture dataset 1 (Supplementary Table 3). For each method we provide the number of sites (N), the Pearson's correlation (r), coefficient of determination (r^2), the Spearman's rank correlation (ρ), and the root mean square error (RMSE) for the comparison of the percentage methylation predicted from Nanopore with the percentage methylation calculated from whole genome bisulfite sequencing (WGBS) data. We show the results for five tested tools and METEORE combining DeepSignal and Megalodon using a random forest (RF) (parameters: max_depth=3 and n_estimator=10) or a regression (REG) model.

	N	*r*	r^2	*ρ*	*RMSE*
Nanopolish	1621	0.8726	0.7614	0.8327	0.2165
DeepSignal	1731	0.9220	0.8500	0.8805	0.1698
Megalodon	1723	0.8786	0.7720	0.8643	0.2336
Tombo	1733	0.8150	0.6642	0.7924	0.2468
Guppy	1733	0.7345	0.5394	0.7275	0.3243
METEORE (RF)	1723	0.9185	0.8437	0.8874	0.1812
METEORE (REG)	1722	0.9167	0.8404	0.8917	0.1866

Supplementary Table 11. Comparison of CpG methylation frequencies from whole genome bisulfite sequencing (WGBS) Illumina data with Cas9-targeted Nanopore data for each method using the double cutoff obtained by discarding 10% of reads. For each site, we removed the 10% of reads with scores closest to the cross point of the FPR and 1-TPR curves in the mixture dataset 1 (Supplementary Table 4). For each method we provide the number of sites (N), the Pearson's correlation (r), coefficient of determination (r^2), the Spearman's rank correlation (ρ), and the root mean square error (RMSE) for the comparison of the percentage methylation predicted from Nanopore with the percentage methylation calculated from whole genome bisulfite sequencing (WGBS) data. METEORE is the combination model with the adjusted parameters of a random forest (max_depth=3 and n_estimator=10) combining DeepSignal and Megalodon.

	N	*r*	r^2	*ρ*	*RMSE*
Nanopolish	2168	0.9376	0.8792	0.8128	0.1641
DeepSignal	2171	0.9677	0.9364	0.8176	0.1187
Megalodon	2171	0.9492	0.901	0.8128	0.1632
Tombo	2171	0.9013	0.8123	0.7809	0.2177
Guppy	2171	0.8867	0.7862	0.7811	0.2248
METEORE (RF)	2171	0.9652	0.9317	0.8274	0.1276
METEORE (REG)	2171	0.9653	0.9318	0.8310	0.1253

Supplementary Table 12. Comparison of CpG methylation frequencies from whole genome bisulfite sequencing (WGBS) Illumina data with Cas9-targeted Nanopore data from Gilpatrick et al. 2020 for each method using the double cutoff obtained by discarding 10% of reads. For each site, we removed the 10% of reads with scores closest to the cross point of the FPR and 1-TPR curves in the mixture dataset 1 (Supplementary Table 4). For each method we provide the number of sites (N), the Pearson's correlation (r), coefficient of determination (r^2), the Spearman's rank correlation (ρ), and the root mean square error (RMSE) for the comparison of the percentage methylation predicted from Nanopore with the percentage methylation calculated from WGBS data. We show the results for five tested tools and METEORE combining DeepSignal and Megalodon using a random forest (RF) (parameters: max_depth=3 and n_estimator=10) or a regression (REG) model.

	No. of CPUs used	Real time per CPU (min)	Peak memory (GB)	Bases per second
Nanopolish	2	10.2	0.2	25433
DeepSignal	9	334.8	23.7	775
Tombo	9	30.4	23.6	8533
Megalodon	11	3258.7	1.4	80
Megalodon (GPU)	1 GPU	3.8 per GPU	2.4	69710
Guppy	11	1494.6	3.3	174
Guppy (GPU)	1 GPU	7.0 per GPU	0.6	37596
DeepMod	8	176.0	2.5	1474
METEORE (RF)	1	0.1	0.3	-
METEORE (REG)	1	1.8	9.5	-

Supplementary Table 13. Runtime and memory usage for each tested tool. We tested each pipeline on the m50 set (2,432 reads and a total of 15,564,827 bases) from the mixture dataset 1. We recorded the real time (wall clock time) from start to finish of the pipeline/command(s) for all tested tools except for METEORE, which took a fast5 directory as an input and output a prediction at genome level (i.e., methylation frequency for each site). For METEORE, we only recorded the time needed for running a single Python script to make the per-read consensus predictions, which was independent of the two methods being combined. Here we used METEORE combining DeepSignal and Megalodon using a random forest (RF) (parameters: max_depth=3 and n_estimator=10) and a regression (REG) model, where both models took per-read prediction outputs generated from the selected tools. To estimate the overall CPU time and memory used, any two tools can be considered, plus the overhead of an additional step of METEORE. We ran each tested tool on a computer with 12 CPU processors (Intel Core i7 (8th Gen) 8700 @ 3.2 GHz). Guppy can be run on CPUs or GPUs, but on a GPU the basecalling speed increase significantly. Megalodon requires Guppy for the basecalling step and uses GPU-enabled Guppy by default. If Megalodon is used with Guppy (CPU), the --guppy-timeout argument should be specified (here we used 200 seconds) to allow sufficient time for calling a read during CPU basecalling. Additionally, we ran Megalodon and Guppy on another computer with a GPU (GeForce RTX 2080 Ti) and 32 CPU processors (AMD Ryzen threadripper 2950x). Real time per CPU = real (wall-clock) time taken x no. of CPUs. Bases per second = total no. of bases/(real time per CPU x 60).
