# Supplementary material for: Systematic benchmarking of tools for CpG methylation detection from nanopore sequencing
Source: Nat Commun. 2021 Jun 8;12:3438. doi: 10.1038/s41467-021-23778-6 (PMC8187371; doi:10.1038/s41467-021-23778-6)
Supplement: Supplementary file 3 — Description of Additional Supplementary Files [file 41467_2021_23778_MOESM3_ESM.pdf]

Description of Additional Supplementary Files

Supplementary Data 1

Filename: Supplementary_data_1.tsv
Description: 100 CpG sites used in mixture dataset 1. A tab separated file including the following fields:

1. Chromosome
2. Ending position

Supplementary Data 2

Filename: Supplementary_data_2.tsv
Description: 50 CpG sites used in mixture dataset 2. A tab separated file including the following fields:

1. Chromosome
2. Ending position

Supplementary Data 3

Filename: Supplementary_data_3.tsv
Description: Fast5 files used to generate each subset in mixture dataset 1. The fast5 files were generated by Simpson et al. (2017) and can be downloaded from the European Nucleotide Archive (ENA) under accession PRJEB13021 (ERR1676719 for negative control and ERR1676720 for positive control). A tab separated file including the following fields:

1. Name of the subset (m0, m10, m20…)
2. Fast5 filename

Supplementary Data 4

Filename: Supplementary_data_4.tsv
Description: Fast5 files used to generate each subset in mixture dataset 2. The fast5 files was generated by Simpson et al. (2017) and can be downloaded from the European Nucleotide Archive (ENA) under accession PRJEB13021 (ERR1676719 for negative control and ERR1676720 for positive control). The reads used in mixture dataset 2 are different from mixture dataset 1. A tab separated file including the following fields:

1. Name of the subset (m0, m10, m20…)
2. Fast5 filename

Supplementary Data 5

Filename: Supplementary_data_5.xlsx

Description: Methylation frequencies of CpG sites calculated by each tested tool for each subset in control mixture dataset 1. An Excel file including the following fields:

1. Chromosome
2. Ending position
3. % Methylated sets
4. Expected methylation frequency
5. Predicted methylation frequency
6. Method
7. Left bound of each 10% methylated bin
8. Right bound of each 10% methylated bin
9. Predicted [inside/outside] a 10% window around the expected methylation proportion

Supplementary Data 6

Filename: Supplementary_data_6.xlsx
Description: The number of true negatives for each tested tool according to different thresholds for the predicted methylation frequency below which a site was called unmethylated, using 0% methylated set from mixture dataset 1. An Excel file including the following fields:

1. Threshold
2. Method
3. Number of true negatives

Supplementary Data 7

Filename: Supplementary_data_7.xlsx
Description: The number of true positives for each tested tool according to different thresholds for the predicted methylation frequency above which a site was called methylated, using 100% methylated set from mixture dataset 1. An Excel file including the following fields:

1. Threshold
2. Method
3. Number of true positives

Supplementary Data 8

Filename: Supplementary_data_8.xlsx
Description: Per-read performance of a binary classifier at all possible classification thresholds for five tested tools measured by receiver operating characteristic (ROC) curve, using reads from 0% methylated set from mixture dataset 1. An Excel file including the following fields:

1. Threshold
2. Specificity, i.e. TN/(TN +FP) = 1−FPR
3. Sensitivity, i.e. TP/(TP/FN) = 1−FNR = TPR

4. FPR
5. Method

, where TP = true positives, FP = false positives, TN = true negatives, FN = false negatives, TPR = true positive rate, and FPR = false positive rate.

Supplementary Data 9

Filename: Supplementary_data_9.xlsx
Description: Per-read performance of a binary classifier at all possible classification thresholds for five tested tools measure by precision-recall (PR) curve, using reads from 0% methylated set from mixture dataset 1. An Excel file including the following fields:

1. Threshold
2. Recall, i.e. TP/(TP/FN) = 1−FNR = TPR
3. Precision, i.e. TP/(TP+FP) = 1−FDR
4. Method

, where TP = true positives, FP = false positives, TN = true negatives, FN = false negatives, TPR = true positive rate, and FDR = false discovery rate.

Supplementary Data 10

Filename: Supplementary_data_10.xlsx
Description: Methylation frequencies of CpG sites calculated by each tested tool for each subset in control mixture dataset 2. An Excel file including the following fields:

1. Chromosome
2. Ending position
3. % Methylated sets
4. Expected methylation frequency
5. Predicted methylation frequency
6. Method
7. Left bound of each 10% methylated bin
8. Right bound of each 10% methylated bin
9. Predicted [inside/outside] a 10% window around the expected methylation proportion

Supplementary Data 11

Filename: Supplementary_data_11.xlsx
Description: Methylation frequencies and coverage of CpG sites reported by each tested tool across the 10 targeted regions from our Cas9-targeted Nanopore sequencing (nCATS) data. An Excel file including the following fields:

1. Chromosome
2. Ending position
3. Methylation frequency reported by Nanopolish
4. Coverage reported by Nanopolish

5. Methylation frequency reported by DeepSignal
6. Coverage reported by DeepSignal
7. Methylation frequency reported by Megalodon
8. Coverage reported by Megalodon
9. Methylation frequency reported by Tombo
10. Coverage reported by Tombo
11. Methylation frequency reported by Guppy
12. Coverage reported by Guppy
13. Methylation frequency reported by DeepMod
14. Coverage reported by DeepMod
15. Methylation frequency reported by METEORE (RF)
16. Coverage reported by METEORE (RF)
17. Methylation frequency reported by METEORE (REG)
18. Coverage reported by METEORE (REG)
19. Methylation frequency reported by whole genome bisulfite sequencing (WGBS)
20. Depth of coverage reported from the BAM file
21. WGBS methylation bin
22. Target region

Supplementary Data 12

Filename: Supplementary_data_12.xlsx
Description: Methylation frequencies and coverage of CpG sites reported by each tested tool across the 8 targeted regions from the Cas9-targeted Nanopore sequencing (nCATS) data generated by Gilpatrick et al. (2020). An Excel file including the following fields:

1. Chromosome
2. Ending position
3. Methylation frequency reported by Nanopolish
4. Coverage reported by Nanopolish
5. Methylation frequency reported by DeepSignal
6. Coverage reported by DeepSignal
7. Methylation frequency reported by Megalodon
8. Coverage reported by Megalodon
9. Methylation frequency reported by Tombo
10. Coverage reported by Tombo
11. Methylation frequency reported by Guppy
12. Coverage reported by Guppy
13. Methylation frequency reported by DeepMod
14. Coverage reported by DeepMod
15. Methylation frequency reported by METEORE (RF)
16. Coverage reported by METEORE (RF)
17. Methylation frequency reported by METEORE (REG)
18. Coverage reported by METEORE (REG)
19. Methylation frequency reported by whole genome bisulfite sequencing (WGBS)
20. Depth of coverage reported from the BAM file
21. WGBS methylation bin

22. Target region

Supplementary Data 13

Filename: Supplementary_data_13.xlsx
Description: 8-mer contexts of the CpG sites and absolute difference between methylation frequencies from Nanopore-based methylation detection tools and whole genome bisulfite sequencing (WGBS) data stratified by different CG content in the 8-mers. Cas9-targeted Nanopore sequencing (nCATS) data from Gilpatrick et al. (2020) were used. An Excel file including the following fields:

1. Chromosome
2. Ending position
3. 8-mer with a CpG in the middle (XXXCGXXX)
4. Method
5. Absolute difference of methylation frequencies between Nanopore-based tools and WGBS
6. Percentage of C and G residues in the 8-mers
7. GC content bin
