# Supplementary material for: Systematic benchmarking of tools for CpG methylation detection from nanopore sequencing
Source: Nat Commun. 2021 Jun 8;12:3438. doi: 10.1038/s41467-021-23778-6 (PMC8187371; doi:10.1038/s41467-021-23778-6)
Supplement: Supplementary file 5 — Reporting Summary [file 41467_2021_23778_MOESM5_ESM.pdf]

nature research

Corresponding author(s): Cameron Jack, Eduardo Eyras

Last updated by author(s): Dec 5, 2021

Reporting Summary

Nature Research wishes to improve the reproducibility of the work that we publish. This form provides structure for consistency and transparency in reporting. For further information on Nature Research policies, see our Editorial Policies and the Editorial Policy Checklist.

Statistics

For all statistical analyses, confirm that the following items are present in the figure legend, table legend, main text, or Methods section.

n/a	Confirmed	
☐	☒	The exact sample size (*n*) for each experimental group/condition, given as a discrete number and unit of measurement
☐	☒	A statement on whether measurements were taken from distinct samples or whether the same sample was measured repeatedly
☐	☒	The statistical test(s) used AND whether they are one- or two-sided<br>*Only common tests should be described solely by name; describe more complex techniques in the Methods section.*
☐	☒	A description of all covariates tested
☐	☒	A description of any assumptions or corrections, such as tests of normality and adjustment for multiple comparisons
☒	☐	A full description of the statistical parameters including central tendency (e.g. means) or other basic estimates (e.g. regression coefficient) AND variation (e.g. standard deviation) or associated estimates of uncertainty (e.g. confidence intervals)
☐	☒	For null hypothesis testing, the test statistic (e.g. *F*, *t*, *r*) with confidence intervals, effect sizes, degrees of freedom and *P* value noted<br>*Give P values as exact values whenever suitable.*
☒	☐	For Bayesian analysis, information on the choice of priors and Markov chain Monte Carlo settings
☒	☐	For hierarchical and complex designs, identification of the appropriate level for tests and full reporting of outcomes
☐	☒	Estimates of effect sizes (e.g. Cohen's *d*, Pearson's *r*), indicating how they were calculated

Our web collection on statistics for biologists contains articles on many of the points above.

Software and code

Policy information about availability of computer code

Data collection	Data was downloaded from public repositories using accessions listed in Data section below. Primary data from the sequencing run was collected from the commercial MinKNOW software (v19.06.8) (Oxford Nanopore Technologies). The raw electrical data in fast5 format was then converted to sequence files in fastq format using the Guppy basecaller (v3.6.0).

Data analysis	All software used for data analysis are fully described in the methods of the manuscript. The custom software - METEORE and all computer code are deposited in GitHub: https://github.com/comprna/METEORE<br><br>Nanopore methylation calling software and versions used:<br>- Nanopolish (v0.13.2)<br>- Tombo (v1.5.1)<br>- DeepSignal (v0.1.7)<br>- Guppy (v3.6.0 used in Guppy pipeline, v4.0.11 used in Megalodon pipeline), v3.2.4 used in post sequencing run basecalling)<br>- Megalodon (v2.2.4)<br>- DeepMod (v0.1.3)<br><br>Additional softwares and versions used:<br>- Minimap2 (v2.17)<br>- Samtools (v1.9)<br>- R (v3.6.3)<br>- weblogo (v3.6.0)<br>- pLogo (v1.2.0)<br>- Integrative Genomics Viewer (v2.6.2)

For manuscripts utilizing custom algorithms or software that are central to the research but not yet described in published literature, software must be made available to editors and reviewers. We strongly encourage code deposition in a community repository (e.g. GitHub). See the Nature Research guidelines for submitting code & software for further information.

Data

Policy information about availability of data

All manuscripts must include a data availability statement. This statement should provide the following information, where applicable:

- Accession codes, unique identifiers, or web links for publicly available datasets
- A list of figures that have associated raw data
- A description of any restrictions on data availability

Nanopore sequencing data generated in this study has been deposited in the Sequence Read Archive (SRA) under study accession PRJNA656260 (https://www.ncbi.nlm.nih.gov/sra/PRJNA656260). Nanopore sequencing data from Gilpatrick et al. used in this study is available in SRA under study accession PRJNA531320 (https://www.ncbi.nlm.nih.gov/sra/PRJNA531320). Nanopore sequencing data for E. coli methylated and unmethylated genomes used in this study is available at the European Nucleotide Archive (ENA) study accession ERP014559 (https://www.ebi.ac.uk/ena/browser/view/PRJEB13021). WGBS data from the ENCODE project18 used in this study is available at https://www.encodeproject.org/ under IDs ENCFF279HCL and ENCFF835NTC. A list of figures that have associated raw data: Figures 2(a,b,c,d), 3(a,b,e,f), 4(a,b,c), 5(a,b,c,d). Source data are also provided with this paper.

Field-specific reporting

Please select the one below that is the best fit for your research. If you are not sure, read the appropriate sections before making your selection.

☒ Life sciences ☐ Behavioural & social sciences ☐ Ecological, evolutionary & environmental sciences

For a reference copy of the document with all sections, see nature.com/documents/nr-reporting-summary-flat.pdf

Life sciences study design

All studies must disclose on these points even when the disclosure is negative.

Sample size	A single sample was sequenced and so this is not applicable.
Data exclusions	"Fail" Nanopore sequencing reads (mean Q-score <= 9) were not used in the analysis.
Replication	As a single sample was sequenced using one MinION flow celll, replication was not required.
Randomization	As a single sample was sequenced, randomization was not required in this study.
Blinding	Blinding was not relevant to this study, as the performance of different tested tools was evaluated by comparing against each other and the ground truth.

Reporting for specific materials, systems and methods

We require information from authors about some types of materials, experimental systems and methods used in many studies. Here, indicate whether each material, system or method listed is relevant to your study. If you are not sure if a list item applies to your research, read the appropriate section before selecting a response.

Materials & experimental systems

n/a	Involved in the study
☒	☐ Antibodies
☐	☒ Eukaryotic cell lines
☒	☐ Palaeontology and archaeology
☒	☐ Animals and other organisms
☒	☐ Human research participants
☒	☐ Clinical data
☒	☐ Dual use research of concern

Methods

n/a	Involved in the study
☒	☐ ChIP-seq
☒	☐ Flow cytometry
☒	☐ MRI-based neuroimaging

Eukaryotic cell lines

Policy information about cell lines

Cell line source(s)	Genomic DNA extracted from GM12878 cell line was used and acquired from Coriell Institute for Medical Research (https://www.coriell.org/0/Sections/Search/Sample_Detail.aspx?Ref=NA12878&product=DNA).
Authentication	No authentication was performed on cell lines.
Mycoplasma contamination	Cultures are tested and found free of mycoplasma contamination by Coriell.
Commonly misidentified lines (See ICLAC register)	No commonly misidentified cell lines were used.
